# Therapeutic mechanism of *Curcuma aromatica* Salisb. rhizome against coronary heart disease based on integrated network pharmacology, pharmacological evaluation and lipidomics

**DOI:** 10.3389/fphar.2022.950749

**Published:** 2022-08-09

**Authors:** Chenghao Fei, De Ji, Huangjin Tong, Yu Li, Lianlin Su, Yuwen Qin, Zhenhua Bian, Wei Zhang, Chunqin Mao, Lin Li, Tulin Lu

**Affiliations:** ^1^ School of Pharmacy, Nanjing University of Chinese Medicine, Nanjing, China; ^2^ Department of Pharmacy, Affiliated Hospital of Integrated Traditional Chinese and Western Medicine, Nanjing University of Chinese Medicine, Nanjing, China; ^3^ Department of Pharmacy, Wuxi Affiliated Hospital of Nanjing University of Chinese Medicine, Wuxi, China; ^4^ School of Pharmacy, Anhui University of Chinese Medicine, Hefei, China

**Keywords:** Curcuma aromatica Salisb. rhizome, coronary heart disease, therapeutic mechanism, network pharmacology, lipidomics, pharmacodynamic substances

## Abstract

*Curcuma aromatica* Salisb. rhizome (CASR) has multifunctional characteristics worldwide and a long history of use as a botanical drug with. Currently, it is often used clinically to treat coronary heart disease (CHD) caused by blood stasis syndrome. However, the therapeutic mechanism of CASR in the treatment of CHD remains poorly understood. In study, the main chemical constituents of CASR were analyzed using UPLC-Q-TOF-MS/MS. Then, its potential therapeutic mechanism against CHD was predicted. Subsequently, pharmacological evaluation was performed using CHD rat model. Finally, a lipidomics approach was applied to explore the different lipid metabolites to verify the regulation of CASR on lipid metabolism disorders in CHD. A total of 35 compounds was identified from CASR. Seventeen active components and 51 potential targets related to CHD were screened by network pharmacology, involving 13 key pathways. *In vivo* experiments showed that CASR could significantly improve myocardial infarction, blood stasis, and blood lipid levels and regulate the PI3K/AKT/mTOR signaling pathway in CHD rats. Lipidomics further showed that CASR could regulate abnormal sphingolipid, glycerophospholipid, and glycerolipid metabolism in CHD rats. The therapeutic mechanism of CASR against CHD was initially elucidated and included the regulation of lipid metabolism. Its effects may be attributed to active ingredients, such as curzerene, isoprocurcumenol, and (+)-curcumenol. This study reveals the characteristics of multi-component and multi-pathway of CASR in the treatment of CHD, which provides a basis for the follow-up development and utilization of CASR.

## 1 Introduction

Coronary heart disease (CHD) is caused by various inducements based on abnormal lipid metabolism (An et al., 2020; Sun et al., 2021). Liquid chromatography-mass spectrometry (LC-MS) was used to detect endogenous metabolic molecular markers in patients with CHD, suggesting severe abnormal lipid metabolism (Cai et al., 2019). Currently, CHD is mainly treated through medication and surgery. The former includes nitrate drugs, antithrombotic drugs, calcium channel blockers, lipid-lowering drugs, and others, while the latter mainly includes percutaneous coronary intervention and coronary artery bypass grafting (Sipahi et al., 2014; Rosendorff et al., 2015).

*Curcuma aromatica* Salisb. rhizome (CASR) is obtained from plants of the genus *Curcuma* of the Zingiberaceae family. These plants are mainly found in the tropics and subtropics of South and Southeast Asia and largely cultivated in Bengal, China, and Sri Lanka (Sun et al., 2017). Although over 100 species reportedly exist worldwide, *Curcuma aromatica* Salisb. is primarily cultivated in China. It promotes blood circulation and reduces pain by regulating qi and relieving blood stasis. It is often used clinically in the treatment of CHD and other diseases (Ding et al., 2015; Jing et al., 2017). CASR mainly contains two categories of components: terpenoids (mainly sesquiterpenes) and curcuminoids (Sun et al., 2017). Modern pharmacological studies have shown that botanical drugs that promote blood circulation and remove blood stasis have many pharmacological effects, such as the regulation of lipid metabolism, dilation of coronary arteries, improvement of microcirculation, and regulation of hemodynamics (Pan et al., 2018). Curcuma oil has been shown to significantly regulate blood lipid indices such as total cholesterol (TC) and triglyceride (TG) and inflammatory factors such as interleukin-2 in atherosclerotic rats, which also provides a clinical basis for CASR in the treatment of CHD caused by atherosclerosis (Singh et al., 2015). However, the pharmacodynamics and therapeutic mechanisms of CASR in CHD treatment remain unclear. Therefore, to further understand the therapeutic mechanism of CASR in the treatment of CHD, we tested a hypothesis that CASR regulates lipid metabolism and improves myocardial infarction through its constituents in the plasma, to treat CHD.

Network pharmacology is a new drug design strategy based on the rapid advances in system biology and multidirectional pharmacology. Lipidomics is an important field of metabolomics. They are widely used to study lipids and their metabolic interactions (Li et al., 2014).

**FIGURE 1:**
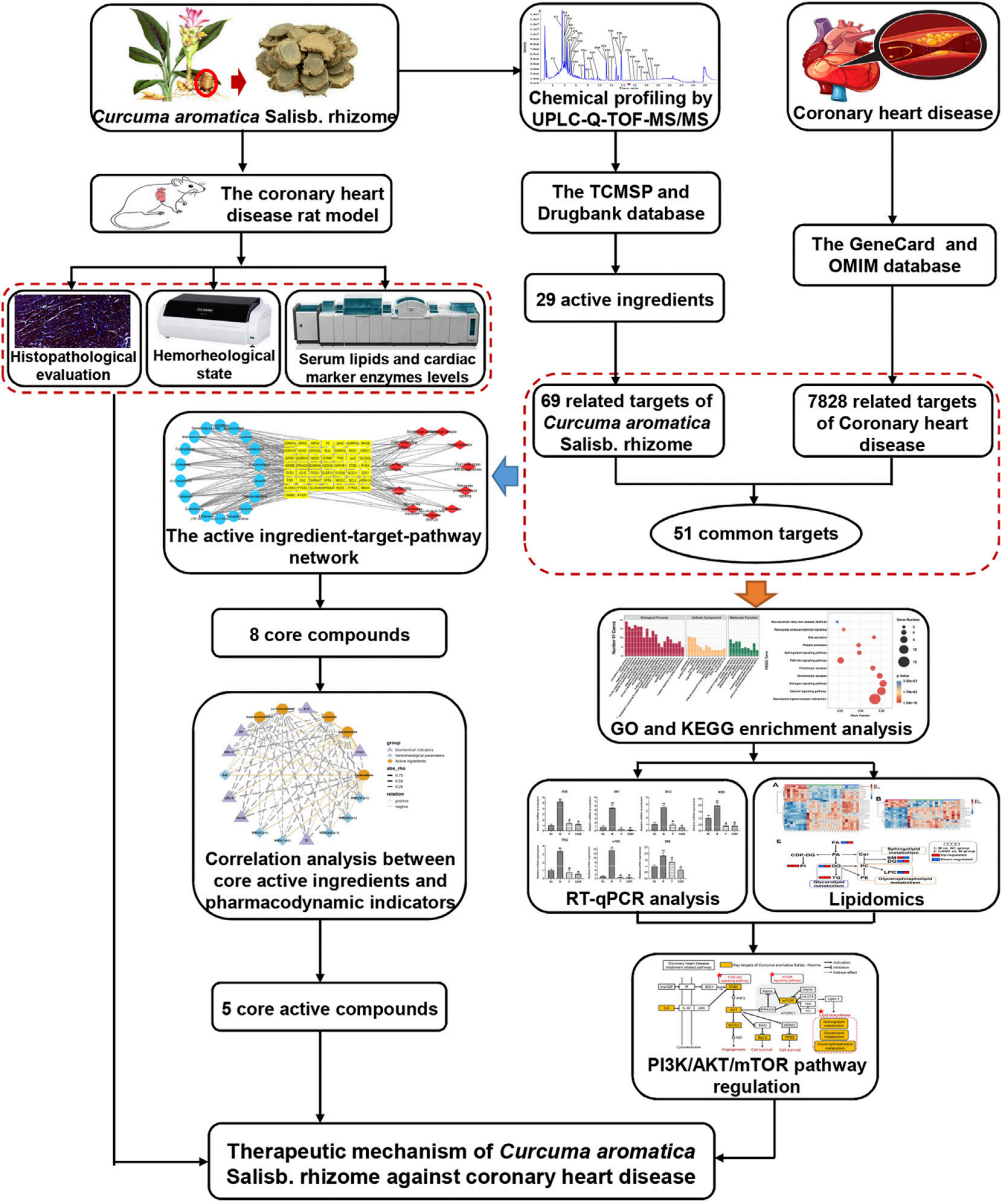
The flowchart of this study.

In this study, first the UPLC-Q-TOF-MS/MS technology was used to analyze the main chemical components of CASR, and then the potential therapeutic mechanism for CHD was predicted by network pharmacology. The therapeutic effect of CASR on CHD was preliminarily evaluated by pharmacological verification, and the expression of the components of PI3K/AKT/mTOR signaling pathway, which was previously predicted, was further verified using reverse transcription-quantitative polymerase chain reaction (RT-qPCR). Finally, a lipidomics approach was applied to reveal the regulation of CASR on lipid metabolism disorders in CHD. The flow chart of this study is shown in Figure 1.

## 2 Materials and methods

### 2.1 Materials and chemicals

The CASR was collected from Zhejiang, China. The samples were identified as rhizomes of *Curcuma aromatica* Salisb. by Professor Jianwei Chen at the Nanjing University of Chinese Medicine. Vouchers for the sample are available at the Nanjing University of Chinese Medicine.

More details could be found in Supplementary File S1.

### 2.2 Chemical profiling of *Curcuma aromatica* Salisb. rhizome

#### 2.2.1 Preparation of sample solution

The CASR slices and extracts were prepared as described in a study (Tong et al., 2021). Finally, CASR extracts containing 1.0 g/ml raw medicine was obtained.

The treatment method for CASR extracts was based on a previously published article (Zhu et al., 2016) with some modifications. After the elution sequence was completed, all eluents were collected and blended adequately. The mixture was centrifuged at 13,000 rpm for 5 min at 4°C, and the supernatant was diluted twice.

#### 2.2.2 Preparation of standard solution

Known amounts of 10 standards (furanodiene, *β*-elemene, demethoxycurcumin, curzerene, germacrone, curcumenol, isocurcumenol, curdione, curcumin, and furanodienon) were weighed accurately and dissolved in methanol to form a mixed stock solution (approximately 5 μg/ml).

#### 2.2.3 Liquid chromatographic and mass spectrometric conditions

Chromatographic and mass spectrometric conditions described in a previous study (Hao et al., 2019) were applied with some modifications. The optimized gradient elution was as follows: 0–1 min, 5–25% B; 1–3 min, 25–30% B; 3–13 min, 30–55% B; 13–15 min, 55–70% B; 15–25 min, 70–100% B; 25–28 min, 100–5% B; 28–30 min, 5% B. The flow rate was 0.3 ml/min and the sample injection volume was 1 µl. In addition, an electrospray ionization (ESI) source (AB SCIEX, CA) with positive mode was used to acquire mass spectra.

#### 2.2.4 Identification of constituents in *Curcuma aromatica* Salisb. rhizome

The raw data were obtained using Analyst TF 1.6 software (AB SCIEX, United States). The methods used for the identification and statistical analysis of compounds were similar to the methods used in a previous article (Cai et al., 2019).

### 2.3 Network pharmacology analysis

#### 2.3.1 Screening of active ingredients

The identified components were screened for absorption, distribution, metabolism, and extraction (ADME) parameters, including oral bioavailability (OB), drug-likeness (DL), and human Caucasian colon adenocarcinoma (Caco-2) permeability, provided by the Traditional Chinese Medicine Systems Pharmacology Database and Analysis Platform (TCMSP, https://tcmspw.com/). The thresholds for OB, DL, and Caco-2 were set to 30%, 0.1, and −0.4, respectively (Wang et al., 2020). In addition, based on the literature, compounds with pharmacological activities were combined.

#### 2.3.2 Acquisition of potential targets of *Curcuma aromatica* Salisb. rhizome against coronary heart disease

TCMSP and DrugBank (https://www.drugbank.com/) were used to search potential gene targets of the active compounds. The species was defined as *homo sapiens*. These target names were converted into official gene symbols using the UniProt database (http://www.uniprot.org/). Duplicates were removed after merging, and the target genes of the active ingredients in CASR were isolated.

The keywords “coronary heart disease” were put into the GeneCards database (http://www.genecards.org) and the Online Mendelian Inheritance in Man database (OMIM) to identify targets derived from the literature and tested using experiments. This process did not set any conditions. After removing duplicates, the related targets of CHD were isolated.

The overlapping targets of the active ingredients in CASR with the related targets of CHD were determined using Venny 2.1 online software (https://bioinfogp.cnb.csic.es/tools/venny/index.html), which were used as potential targets of CASR against CHD.

#### 2.3.3 Gene ontology and kyoto encyclopedia of genes and genomes enrichment analysis

Gene ontology (GO) and Kyoto Encyclopedia of Genes and Genomes (KEGG) pathway analyses of the potential targets of CASR against CHD were performed using the Metascape database (http://metascape.org/). Official gene symbols were used and *homo sapiens* was selected. GO enrichment terms included biological processes (BP), molecular functions (MF), and cellular components (CC). A threshold of *p* < 0.01 was set, and the targets were sorted according to the gene number involved. OmicStudio (https://www.omicstudio.cn/tool) was used to screen and plot top-ranking biological processes and pathways.

#### 2.3.4 Construction of the active ingredient-target-pathway network

The active ingredients, potential targets of CASR against CHD, and the KEGG pathways were imported to the Cytoscape (Version 3.8.2) software for the interaction network construction. Additionally, the CytoNCA plug-in was used to calculate topological parameters including “Degree,” “Closeness Centrality,” and “Betweenness Centrality”.

### 2.4 Pharmacological evaluation of *Curcuma aromatica* Salisb. rhizome against coronary heart disease *in vivo*

#### 2.4.1 Animals and grouping

Male specific pathogen-free Sprague-Dawley rats (180–200 g) were obtained from the Experimental Animal Center, Hangzhou Medical College, Zhejiang, China (license number: SCXK (ZHE) 2019-0002). The animals were kept in a temperature (25 ± 2°C), humidity (60% ± 5%), and daylight (periodic 12 h light/dark cycle)-controlled room but with free access to standard diet and water. Following 1 week of acclimatization, the rats were divided randomly into a normal control group (NC) (*n* = 8), model group (M) (*n* = 15), positive drug (atorvastatin calcium tablets) control group (P) (*n* = 8), CASR high-dose treatment group (CASR-H) (*n* = 8), and CASR low-dose treatment group (CASR-L) (*n* = 8).

#### 2.4.2 Experimental protocol and drug administration

The CHD model group of rats was fed an HFD combined with vitamin D_3_ and isoproterenol hydrochloride. The modeling method followed that of a previous study (Sun et al., 2019) with some modifications. The experimental period was 17 weeks. The NC group was fed a normal diet, and the other groups were fed an HFD. On the first day of modeling, except for the NC group, the other groups were orally administered with vitamin D_3_ (600,000 IU/kg). Additional vitamin D_3_ (100,000 IU/kg) was administered orally at the 2nd, 4th, 6th, and 8th week, and the same volume of normal saline was administered to the rats in the NC group. For the last 2 days of the 17th week, 30 min after oral administration, M, P, and CASR group of rats were subcutaneously injected (on the back) with isoproterenol hydrochloride (85 mg/kg), once each day. The NC group received the same volume of normal saline. The experimental protocol was in accordance with the Regulations of Experimental Animal Administration issued by the State Committee of Science and Technology of the People’s Republic of China.

From week 15 of modeling, the drug administration scheme was as follows: CASR-L group was orally administered with CASR extracts at a dose of 0.63 g/kg/d; CASR-H group was orally administered with CASR extracts at a dose of 0.95 g/kg/d; P group was orally administered with atorvastatin calcium tablets at a dose of 2.1 mg/kg/d; other groups were orally administered with the same amount of normal saline. The CASR dosage was converted equally according to the upper and lower limits stipulated in the Chinese Pharmacopoeia (2020 edition) and referred to previous article (Xie et al., 2020).

During the experimental period, six rats died. Among the 41 rats that survived, 8, 13, 7, 7, and 6 rats belonged to the NC, M, P, CASR-L, and CASR-H groups, respectively. The modelling rate exceeded 84%.

#### 2.4.3 Sample collection and detection

The rats were euthanized 30 min after the final dose. Serum and plasma samples were immediately collected using the abdominal aortic method. Hemorheological parameters, such as whole blood viscosity (WBV) (200 s^−1^, 30 s^−1^, 5 s^−1^, 1 s^−1^) and erythrocyte aggregation index (EAI), were measured using the SA-9000 Automated Blood Rheology Analyzer (Beijing Succeeder, China). Serum TC, high-density lipoprotein cholesterol (HDL-C), and low-density lipoprotein cholesterol (LDL-C) levels were determined using Roche Cobas 8000 c702 (Roche Diagnostics, United States). Interleukin-6 (IL-6), oxidized low-density lipoprotein (OxLDL), endothelin (ET), and cardiac troponin I (cTn-I) levels in the serum were determined using the ELISA kits. Plasma was stored at −80°C for lipidomic analysis. Simultaneously, the heart was soaked in 4% PFA and embedded in paraffin. Hematoxylin and eosin (H&E) and Masson’s trichrome (MT) were applied to each of sections (5 µm thick). The histopathological samples were then examined under a light microscope (Leica, DFC-259, Germany).

#### 2.4.4 Real-time quantitative PCR

The expression levels of PI3K, AKT, Bcl-2, TP53, NOS3, mTOR and SMS in the myocardial tissue were detected using RT-qPCR (Chen et al., 2022). *β*-Actin was the reference gene. Relative mRNA expression was calculated using 2^−ΔΔCT^ method. Supplementary Table S1 lists the sequences of all primers.

### 2.5 Lipidomics analysis

#### 2.5.1 Sample preparation

A plasma sample preparation strategy was employed based on a previous study (Shan et al., 2018). Internal standards (lysoPE (17:1), D5 TG (17:0–17:1–17:0), and PE (17:0), concentrations of approximately 2.5 μg/ml) were used. Finally, the supernatant was used for the analysis. Quality control (QC) samples were prepared simultaneously to ensure that the method and system were suitable.

#### 2.5.2 Liquid chromatographic and mass spectrometric conditions

Lipidomics analysis conditions were based on a previous study (Shan et al., 2018) with some modifications. The optimized gradient elution was as follows: 0–2 min, 15–30% B; 2–2.5 min, 30–48% B; 2.5–11 min, 48–82% B; 11–11.5 min, 82–99% B; 11.5–12 min, 99% B; 12–13 min, 99–15% B; 13–15 min, 15% B. The column oven temperature was maintained at 65°C. The flow rate was 0.6 ml/min, and the sample injection volume was 3 µl (positive mode) and 2 µl (negative mode). An electrospray ionization (ESI) source was used for both the positive and negative modes. The scan range was 215–1800 m/z.

#### 2.5.3 Lipid annotation and data processing

Lipid annotation and data processing methods were used, as described in previous studies (Shan et al., 2018; Yang et al., 2019). MetaboAnalyst 5.0 (https://www.metaboanalyst.ca/) was used for the multivariate analysis. Based on the logarithmic transformation (base 10) and Pareto scaling, statistical models were developed for the total ion chromatogram (TIC) normalized data (SERRF). Partial least-squares discriminant analysis (PLS-DA) was used to develop the classificatory/predictive models. Two factors were combined to select the significantly different lipids: (I) *p* value <0.05; (II) variable importance in projection (VIP) value >1.0.

### 2.6 Statistical analysis

Statistical analysis was performed using the SPSS software (version 23.0; IBM, United States). The results were expressed as mean ± standard deviation (SD). Differences between groups were analyzed using unpaired T tests. The significance level was set at *p* < 0.05.

## 3 Results

### 3.1 Constituent analysis in *Curcuma aromatica* Salisb. rhizome

A typical TIC of the compounds in CASR is shown in Supplementary Figure S1. Through a comparison with the standards and mass spectral fragmentation pathways in the literature, 35 compounds were identified in the CASR. Detailed information on the identified compounds can be found in Supplementary Table S2. These compounds mainly contained two monoterpenes, 31 sesquiterpenes (13 guaiane-type, 2 carabrane-type, 5 elemane-type, 10 germacrane-type, and 1 unique type), and two curcuminoids.

### 3.2 Prediction of the therapeutic mechanism of *Curcuma aromatica* Salisb. rhizome against coronary heart disease

#### 3.2.1 Active compounds in *Curcuma aromatica* Salisb. rhizome

According to ADME screening, 19 candidate compounds were obtained. In addition, 10 compounds with pharmacological activities were combined. For example, curcumin (P23, OB = 4.37%, DL = 0.41, and Caco-2 = 0.35) could reduce lipid levels in the blood and inhibit CHD progression (Li et al., 2015), despite its relatively low OB value. A total of 29 candidate active compounds were screened (Table 1) for further analyses.

**TABLE 1:** Potential active compounds of CASR in the treatment of CHD and their ADME parameters.

No.	RT (min)	Compound	OB (%)	DL	Caco-2	MOL ID
P2	3.71	Gweicurculactone	42.92	0.14	1.37	MOL000903
P3	4.11	Zedoalactone A	111.43	0.19	-0.01	MOL004305
P4^*^	4.15	Zedoarolide B	135.56	0.21	-0.42	MOL004313
P6	4.25	Zedoalactone C	72.82	0.17	-0.21	MOL004307
P7	4.32	Curcumenone	34.17	0.11	0.91	MOL000898
P8	4.39	Wenjine	47.93	0.27	0.30	MOL000906
P9	4.74	(1R,10R)-Epoxy-(-)-1,10-dihydrocurdione	36.73	0.12	0.68	MOL000891
P10	4.78	Curzerenone	57.05	0.11	1.28	MOL000900
P11	4.82	Isoprocurcumenol	46.11	0.10	0.60	MOL000949
P12	4.90	Zedoalactone B	103.59	0.22	-0.08	MOL004306
P14	5.43	Aerugidiol	38.70	0.12	0.10	MOL000897
P16	7.45	Procurcumenol	34.40	0.10	0.67	MOL000961
P17	8.23	(4S)-Dihydrocurcumenone	64.35	0.11	0.76	MOL004327
P18	8.95	Neocurcumenol	87.75	0.13	1.11	MOL004286
P19	9.66	4-epi-Curcumenol	89.38	0.13	1.06	MOL004326
P20^*^	11.27	Furanodiene	45.11	0.10	1.77	MOL000899
P21	11.27	(+)-Curcumenol	87.82	0.13	1.14	MOL000901
P22^*^	11.51	Demethoxycurcumin	4.37	0.33	0.31	MOL000946
P23^*^	12.02	Curcumin	4.37	0.41	0.35	MOL000892
P24^*^	12.86	Neocurdione	36.65	0.08	0.88	MOL004287
P25^*^	13.72	β-Elemenone	28.12	0.07	3.79	MOL004337
P26^*^	13.72	Curdione	7.00	0.08	0.81	MOL000896
P27^*^	13.73	(+)-Limonene	39.84	0.02	1.83	MOL000023
P28^*^	15.30	Curzerene	47.86	0.09	1.74	MOL004258
P29	16.01	Furanodienon	44.67	0.11	1.18	MOL001172
P30	16.41	Curcumol	103.55	0.13	1.12	MOL000902
P31	17.38	Isocurcumenol	97.67	0.13	1.11	MOL000889
P33^*^	18.74	Germacrone	32.50	0.07	1.33	MOL000910
P34^*^	19.48	β-Elemene	25.63	0.06	1.84	MOL000908

“^*^” active compounds from literatures.

#### 3.2.2 Potential targets of *Curcuma aromatica* Salisb. rhizome against coronary heart disease

Overall, 18 of the 29 candidate active compounds yielded 69 targets, according to TCMSP and DrugBank. Furthermore, 7,716 and 200 targets related to CHD were collected using GeneCards and OMIM, respectively. A total of 7,828 targets were collected after merging and removing the duplicates. The interaction of the targets of the active ingredients in CASR and CHD was considered the potential target of CASR against CHD. A total of 51 shared targets were identified.

#### 3.2.3 GO and KEGG enrichment analysis

**FIGURE 2:**
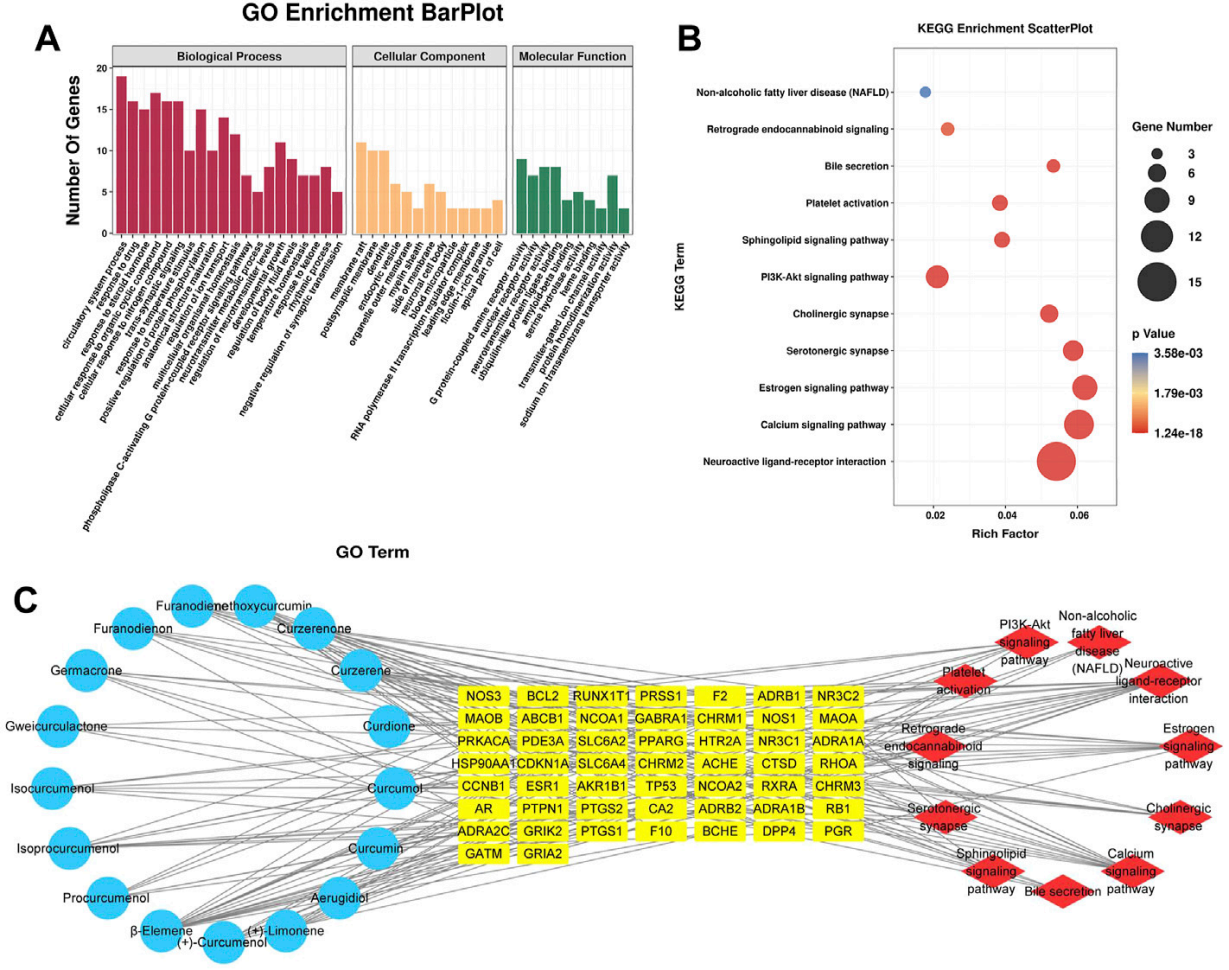
Analysis results of network pharmacology. **(A)** GO enrichment analysis. The horizontal axis represents the GO term, and the vertical axis represents the number of genes; **(B)** KEGG pathway enrichment analysis. The horizontal axis represents the rich factor, and the vertical axis represents the pathways. The bubble size represents the number of targets in the pathway. The bubble color indicates the magnitude of the *p*-value. The redder the color, the lower the *p*-value; **(C)** The active ingredient-target-pathway network. Blue represents the active components, yellow represents targets, and red represents the pathways.

The 51 potential target genes of CASR against CHD were enriched and analyzed using the Metascape database. The results of GO and KEGG pathway analyses are shown in Figure 2. There were 33 main GO terms. Enriched biological processes included the circulatory system process, response to drug, response to steroid hormone, cellular response to organic cyclic compounds, and cellular response to nitrogen compounds; molecular functions included G protein-coupled amine receptor activity, nuclear receptor activity, neurotransmitter receptor activity, ubiquitin-like protein ligase binding, and amyloid-beta binding; cellular components included membrane rafts, postsynaptic membranes, dendrites, and endocytic vesicles (Figure 2A). KEGG pathway analysis indicated that CASR exerted regulatory effects through several pathways, including neuroactive ligand-receptor interaction, calcium signaling pathway, estrogen signaling pathway, serotonergic synapse, cholinergic synapse, PI3K-Akt signaling pathway, sphingolipid signaling pathway, platelet activation, bile secretion, retrograde endocannabinoid signaling, and non-alcoholic fatty liver disease (Figure 2B).

#### 3.2.4 Construction of the interaction network

Based on the results of the analysis, we predicted the specific mechanism of CASR against CHD. A global view of the active ingredient-target-pathway network, including 79 nodes (17 compounds, 51 potential targets, and 11 signaling pathways) and 197 edges, was constructed (Figure 2C). A complex relationship existed among the active ingredients in CASR, potential target genes, and the KEGG pathways related to them. Active ingredients, targets, pathways with degree, closeness centrality, and betweenness centrality ≥ their median values were screened as core elements of CASR against CHD. Finally, 8 core ingredients, 21 core targets, and 5 core pathways were screened. The results are listed in Table 2. The highest ranked compound was *β*-elemene. Certain target genes were found to be linked to various pathways. For example, *CHRM1* participates in the neuroactive ligand-receptor interaction pathway, calcium signaling pathway, cholinergic synapse, and PI3K-Akt signaling pathway, whereas *GABRA1* is involved in the neuroactive ligand-receptor interaction and retrograde endocannabinoid signaling pathway. These results suggest that CASR acts on CHD through a multitarget, multipathway, and integrative manner.

**TABLE 2:** Core ingredients, targets and pathways of CASR against CHD.

Categories	No.	Name	Degree	Closeness centrality	Betweenness centrality
Ingredients	1	β-Elemene	17	0.4407	1158.1643
	2	Curzerene	13	0.4041	477.4343
	3	Demethoxycurcumin	10	0.3768	721.6091
	4	Curzerenone	9	0.3697	430.3524
	5	Isoprocurcumenol	9	0.3498	267.5783
	6	Furanodiene	8	0.3768	199.6757
	7	(+)-Curcumenol	7	0.3498	474.8281
	8	(+)-Limonene	6	0.3805	159.5248
Targets	1	CHRM1	12	0.4239	449.3520
	2	GABRA1	12	0.4382	877.9484
	3	PTGS2	11	0.4021	484.3543
	4	CHRM2	10	0.4021	322.9952
	5	PRKACA	9	0.3939	461.0935
	6	CHRM3	8	0.3900	203.9512
	7	PTGS1	8	0.3786	272.0650
	8	NOS3	7	0.3679	217.8825
	9	ADRB2	6	0.3714	228.0657
	10	F2	6	0.3611	144.2236
	11	NR3C1	5	0.3391	255.5735
	12	BCL2	5	0.3451	102.1192
	13	NCOA2	5	0.3514	162.6703
	14	RXRA	5	0.3545	171.7665
	15	ADRA1B	4	0.3421	37.9374
	16	ADRA1A	4	0.3645	55.2813
	17	HTR2A	4	0.3421	51.9311
	18	HSP90AA1	4	0.3333	101.4216
	19	PGR	4	0.3223	70.3906
	20	SLC6A2	4	0.3482	48.3808
	21	RHOA	3	0.3223	33.3751
Pathways	1	Neuroactive ligand-receptor interaction	15	0.4171	769.3469
	2	Calcium signaling pathway	11	0.3959	349.4705
	3	Estrogen signaling pathway	9	0.3732	474.9934
	4	PI3K-Akt signaling pathway	8	0.3732	199.6417
	5	Cholinergic synapse	6	0.3697	132.2672

### 3.3 Pharmacological verifications

#### 3.3.1 Effect of *Curcuma aromatica* Salisb. rhizome on cardiac changes in coronary heart disease rats

**FIGURE 3:**
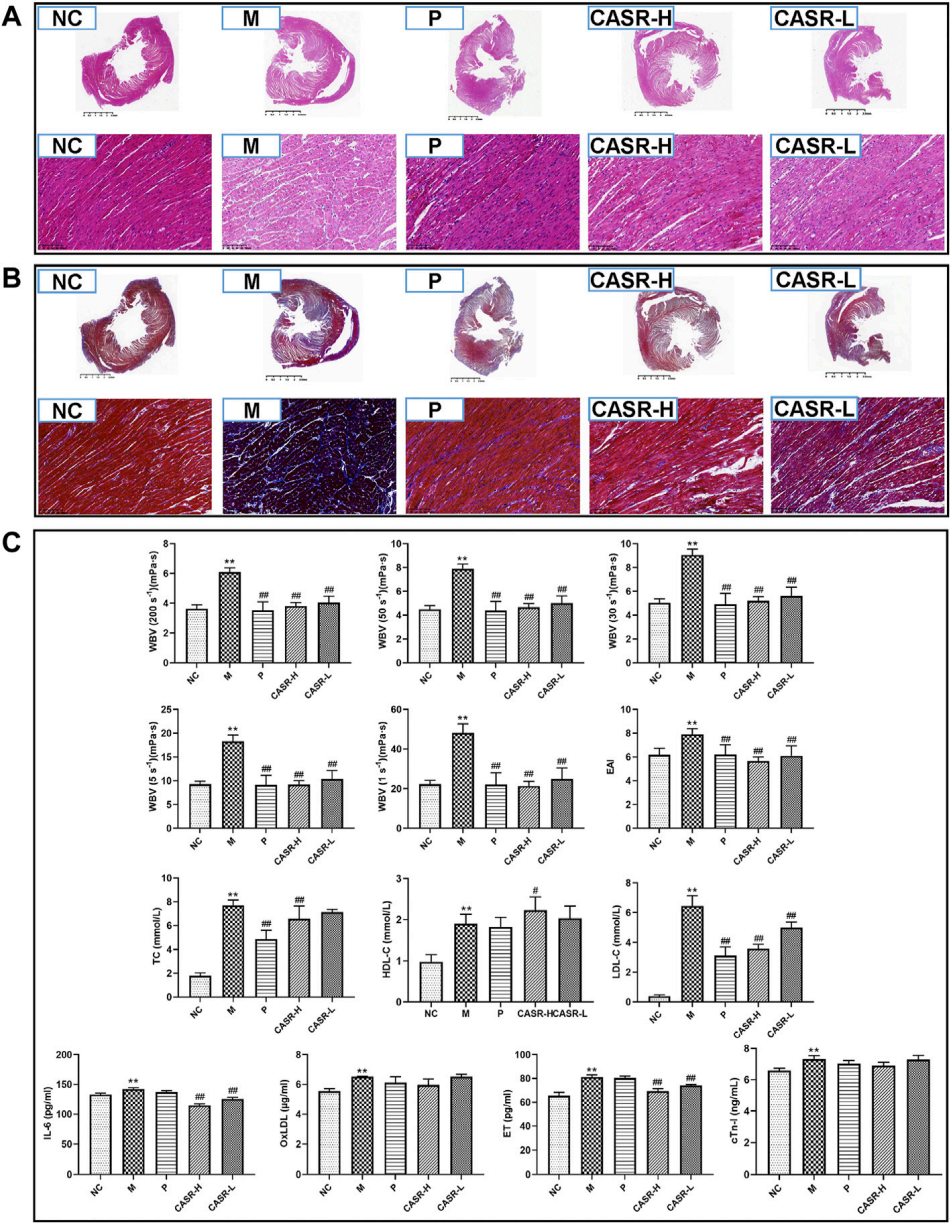
Experimental validation results *in vivo*. **(A)** Heart histopathological changes of H&E staining (magnification = 20× and 500×). Nucleus was stained purple-blue and cytoplasm was stained red; **(B)** Heart histopathological changes of Masson staining (magnification = 20× and 500×). Collagen fibers appear in blue and muscle fiber appear in red; **(C)** Hemorheological parameters and serum biochemical indicators. All values were presented as mean ± SD (n = 6–8). All *p*-values were calculated by T test. ^*^ *p* < 0.05, ^**^ *p* < 0.01 compared with NC group, ^#^ *p* < 0.05, ^##^ *p* < 0.01 compared with M group. NC: normal control group; M: model group; P: positive drug (atorvastatin calcium tablets) control group; CASR-H: *Curcuma aromatica* Salisb. rhizome high-dose treatment group; CASR-L: *Curcuma aromatica* Salisb. rhizome low-dose treatment group.

Histopathological characteristics of CHD rats are shown in Figures 3A,B. In the NC group, the cardiomyocytes were dense and arranged neatly, with nuclei of the same size and shape. A normal space was observed in the myocardium. No inflammatory cell infiltration or myocardial fibrosis was observed. However, in the M group, the arrangement of cardiomyocytes was disordered, and the nuclei were deeply stained. The myocardial space had widened. There was a large amount of degeneration and necrosis of the myocardial fibers with inflammatory cell infiltration. Meanwhile, there was a significant increase in the amount of blue-stained collagen fibers, and extensive fibrosis were observed. In contrast, the degree of myocardial fibrosis, necrosis, and inflammatory cell infiltration in the P group was significantly lower than that of the model group. The therapeutic effect of the CASR groups was close to that of the P group and that of the CASR-H group was more significant.

#### 3.3.2 Effect of *Curcuma aromatica* Salisb. rhizome on hemorheological parameters in coronary heart disease rats

The hemorheological parameters of rats are shown in Figure 3C. The WBV and EAI were significantly higher in the M group (*p* < 0.01) than in the NC group, indicating that the rats in the M group were in a serious state of blood stasis. However, the hemorheological parameters of the rats in each treatment group decreased significantly (*p* < 0.01). The results of the P group were close to that of the NC group and that of the CASR-H group was more significant.

#### 3.3.3 Effect of *Curcuma aromatica* Salisb. rhizome on serum biochemical indicators in coronary heart disease rats

The results for serum lipids and cardiac marker enzymes are summarized in Figure 3C. Serum concentrations of TC, HDL-C, LDL-C, IL-6, OxLDL, ET, and cTn-I were higher in the M group than in the NC group. Except for HDL-C, all other indices in each CASR-administered group showed a downward trend in comparison to those in the M group. Most of the changes were significant, and the curative effect of CASR was comparable to that of the positive drug.

#### 3.3.4 Effect of *Curcuma aromatica* Salisb. rhizome on the PI3K/AKT/mTOR signaling pathway

To further investigate the mechanism of CASR in the treatment of CHD, the combined results of the KEGG enrichment analysis in network pharmacology prediction and the expression analysis of PI3K/AKT/mTOR signaling pathway components was verified. The expression of the key genes PI3K, AKT, Bcl-2, TP53, NOS3, and mTOR in this pathway was detected using RT-qPCR. The mTOR pathway was related to lipid metabolism. As shown in Supplementary Figure S2, CASR significantly downregulated the expression of PI3K, AKT, Bcl-2, TP53, NOS3, mTOR, and SMS in CHD rats.

### 3.4 Lipidomics analysis

#### 3.4.1 Data quality assurance

The method was validated as described in a previous article (Yang et al., 2019). The relative standard deviations of the peak areas of the lipid internal standards were less than 15.83%, indicating that the experimental operation and instrument conditions were relatively stable.

#### 3.4.2 Identification of lipid compounds

TICs of the samples from the positive and negative ion modes are shown in Supplementary Figure S3. The lipids were identified using MS-DIAL software and the LipidBlast database.

In the positive ion mode, 422 lipids were identified, mainly containing acylcarnitine (CAR), cholesteryl ester (CE), and diacylglycerol (DG). In the negative ion mode, 154 lipids were found, mainly containing bile acid (BA), ceramide (Cer), and cardiolipin (CL). To summarize, 576 lipids from plasma samples were identified.

#### 3.4.3 Metabolic profile analysis of lipids

The datasets of each group were analyzed using PLS-DA, and each point in the figure represents a sample. As shown in Supplementary Figure S4A and Supplementary Figure S5B, the NC and M groups displayed significant separation, while CASR had a restorative effect, especially in the positive ion mode. The permutation test results showed that the PLS-DA model was not over-fitted (*p* < 0.05), validating the model (Supplementary Figure S4B; Supplementary Figure S5B). In addition, Supplementary Figure S4C; Supplementary Figure S5C showed obvious separation of samples between the NC and M groups. Supplementary Figure S4D; Supplementary Figure S5D showed same separation between the M and CASR-administered groups.

#### 3.4.4 Comparative analysis of differential lipids

**FIGURE 4:**
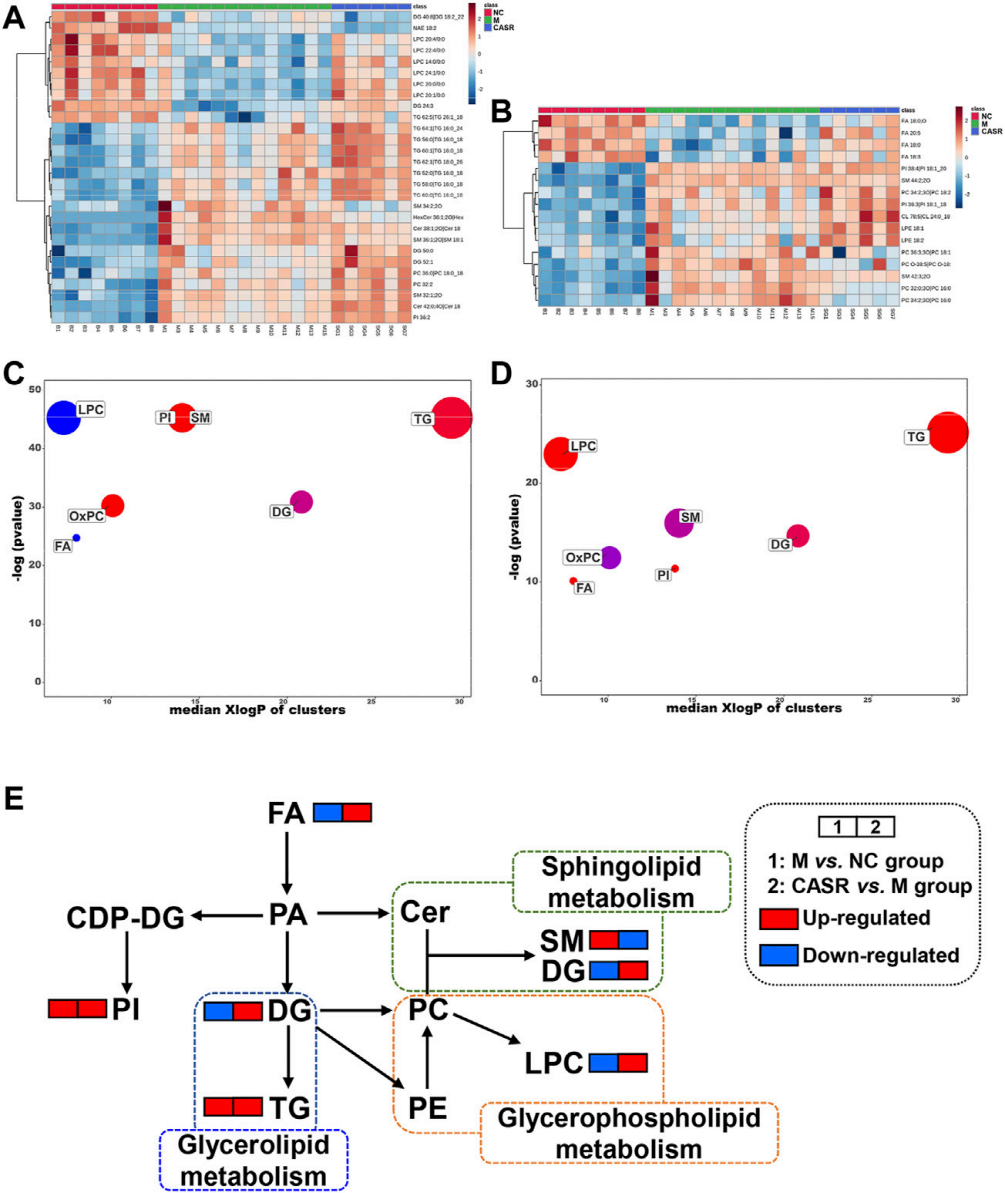
Summary of differential lipids. **(A)** Heatmap of differential lipids in positive mode; **(B)** Heatmap of differential lipids in negative mode. Each column represents a sample and each row represents a differential lipid. Red changes to blue when the intensity becomes smaller, and vice versa. The darker the red, the larger the value; **(C)** Enrichment statistics plot of significantly regulated lipids (M vs. NC group); **(D)** Enrichment statistics plot of significantly regulated lipids (CASR vs. M group). Each bubble represented a significantly changed lipid group (*p* < 0.05), and the bubble size reflected the total number of lipids contained in each lipid group. Red represents increased lipids and blue represents decreased lipids. The purple bubbles have both increased and decreased lipids; **(E)** The metabolic network profile. The names of the possible metabolic pathways are denoted in the green, blue and orange dotted box. NC: normal control group; M: model group; P: positive drug (atorvastatin calcium tablets) control group; CASR: *Curcuma aromatica* Salisb. rhizome treatment group.

A total of 265 differential lipids with the criteria of *p* < 0.05 and VIP >1.0 were (NC group vs. M group) screened. Among them, 44 were regulated after CASR administration (28 in the positive ion mode and 16 in the negative ion mode). The 44 differentially expressed lipids are shown in Supplementary Table S3. Heat maps are also used to visually show the differences in lipids among the groups (Figures 4A,B).

In addition, enrichment analysis was beneficial for obtaining mechanistic insights into the lipids. As shown in Figures 4C,D, the seven lipid classes in the M group were significantly different from those in the NC group. Except for the downregulation of LPC and FA, the trend for other lipid classes was upward as a whole. After CASR treatment, SM, LPC, FA, and OxPC levels were regulated (*p* < 0.05). SM 34:2; 2O was the most downregulated lipid in SM, LPC 20:1/0:0 was the most upregulated lipid in LPC, FA 20:5 was the most upregulated lipid in FA, and PC 32:0; 3O|PC 16:0_16:0; 3O was the most downregulated lipid in OxPC.

Further analysis performed on the metabolic network (Figure 4E) to understand the interaction between different metabolites showed that CASR in the CHD treatment mainly regulated the sphingolipid metabolism, glycerophospholipid metabolism, and glycerolipid metabolism.

### 3.5 Correlation analysis between core active ingredients and pharmacodynamic indicators

**FIGURE 5:**
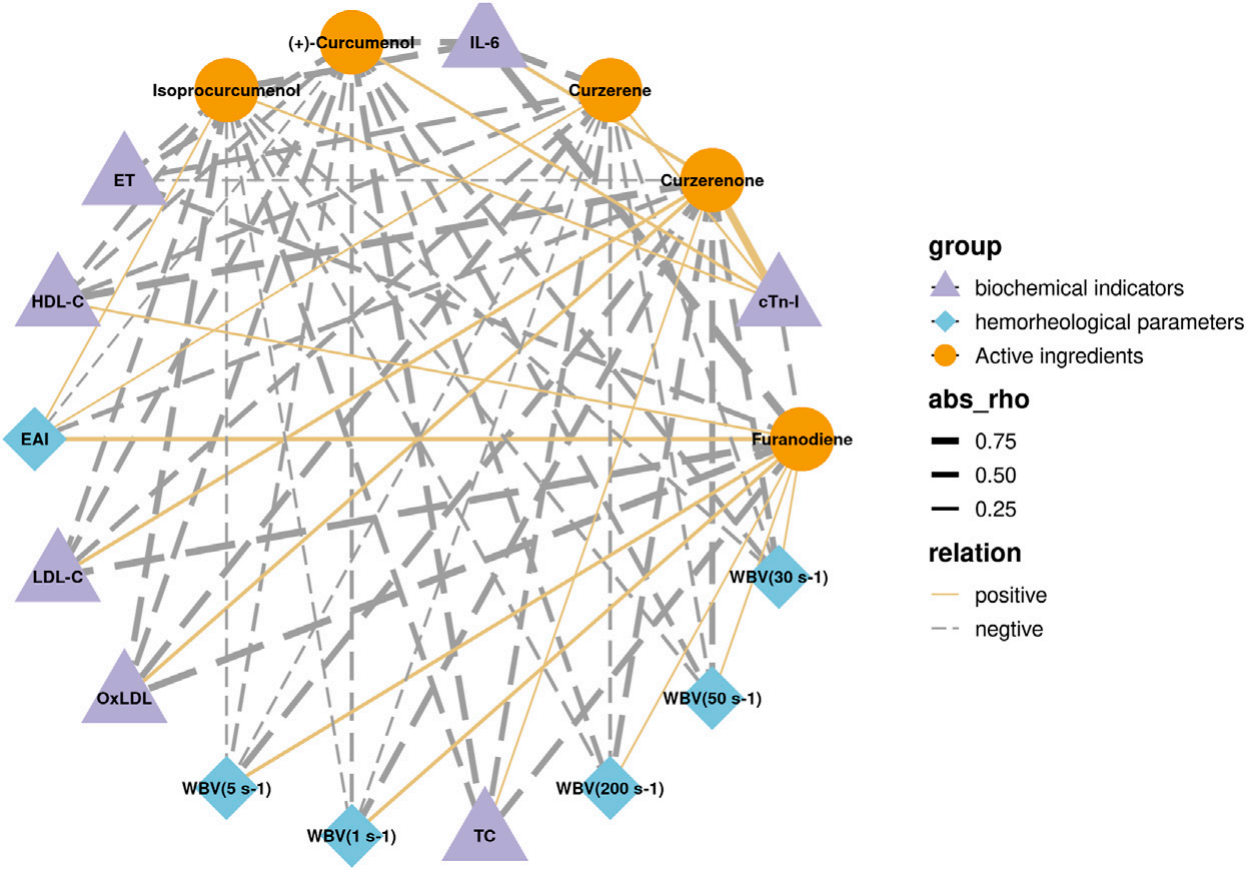
Correlation analysis results between core active ingredients and pharmacodynamic indicators. Purple triangles represent biochemical factors; blue diamonds represent hemorheological parameters; yellow circles represent active compounds; the thickness of the line represents the magnitude of the correlation; solid and dashed line represents positive and negative correlations, respectively.

Among the eight core compounds listed in Table 2, only five were detected in the plasma of the CASR treatment groups. These active compounds were then correlated with pharmacodynamic indicators, such as hemorheological parameters and biochemical indicators. The *in vivo* detection and analysis methods for compounds in CASR were based on our previous study (Tong et al., 2021). The correlation network is shown in Figure 5. Curzerene, isoprocurcumenol, and (+)-curcumenol were negatively correlated with most pharmacodynamic indicators, including WBV, TC, and ET. Furanodiene was negatively correlated with the biochemical indicators. Curzerenone was negatively correlated with hemorheological parameters and positively correlated with biochemical indicators. *β*-elemene, demethoxycurcumin, and (+)-limonene were not detected.

## 4 Discussion

In the network pharmacology analysis, GO enrichment analysis revealed that CASR against CHD is involved in a number of biological processes, such as circulatory processes, response to steroids, and phospholipase C activating G protein-coupled receptor signaling pathway, which are directly related to the pathogenesis of CHD (Jayalalitha et al., 2008; Morgan et al., 2020; Nagoor et al., 2020). The KEGG enrichment analysis also suggested that CASR regulates CHD through a variety of pathways. These pathways were partially confirmed by experiments, such as the neuroactive ligand-receptor interaction pathway (Wang et al., 2014), calcium signaling pathway, estrogen signaling pathway (Fortini et al., 2019), PI3K-Akt signaling pathway (Wang et al., 2019), sphingolipid signaling pathway and bile secretion. These results indicated that CASR in the treatment of CHD could protect nerves, repair vascular smooth muscle necrosis, alter lipid metabolism, and reduce inflammation.

In the “active ingredient-target-pathway” network, according to the order of the active compounds involved, there were eight compounds, each of which with a degree greater than the median. The core active ingredients *β*-elemene, curzerene, demethoxycurcumin, curzerenone, isoprocurcumenol, furanodiene, (+)-curcumenol, and (+)-limonene, interacted on an average with 10 targets. They have been shown to exert protective effects against cardiovascular diseases. For example, furanodiene, demethoxycurcumin, and (+)-curcumenol exhibit anti-inflammatory activities (Makabe et al., 2006; Li et al., 2019; Pintatum et al., 2020). Curzerenone and isoprocurcumenol inhibit platelet aggregation through the regulation of the MAKR and PI3K/AKT signaling pathways (Tong et al., 2021). The *β*-elemene could improve atherosclerosis by restoring nitric oxide (NO) levels and alleviating oxidative stress, which is associated with CHD (Liu et al., 2017). It is worth noting that although curcuminoids have anti-inflammatory, antioxidant, and hypolipidemic activities, they have poor solubility and low bioavailability, unless the drug delivery mode can be changed (Tagde et al., 2021). Previous studies have shown that furanodiene can induce apoptosis by caspase and p38 MAPK activation, and this process is also accompanied by the inhibition of Bcl-XL, AKT, Bad, and Bax (Aggarwal et al., 2013). Curcumenol significantly reduced LPS-induced production of NO and proinflammatory cytokines (IL-6 and TNF-α) and the expression of proinflammatory proteins iNOS and COX-2, which are associated with AKT-mediated inhibition of the NF-KB and p38 MAPK signaling pathways (Lo et al., 2015). The above mechanisms are the anti-inflammatory mechanisms of furanodiene and (+)-curcumenol respectively.

Each target interacts with an average of two compounds. For example, CHRM1 interacts with (+)-limonene, curzerene, furanodiene, germacrone, isoprocurcumenol, isocurcumenol, *β*-elemene, and curdione. Research has shown that CHRM1 plays a critical role in regulating atrial repolarization in I_K, Ach_. Furthermore, CHRM1 and CHRM2 regulate K^+^ channels through the action of G proteins. At the same time, CHRM1 increases the heart rate and contractility (Tai et al., 2019). Each signaling pathway was regulated by an average of seven targets. The PI3K/AKT pathway is an important intracellular signal transduction pathway that mediates a variety of biological effects, including regulation of vascular endothelial formation, inflammatory response, cardiomyocyte apoptosis, and autophagy, as well as the downstream mTOR signaling pathway to regulate lipid metabolism, which has a substantial impact on the development of cardiovascular diseases. Through the active ingredient-target-pathway network, it was found that the PI3K/AKT signaling pathway was enriched by eight targets such as NOS3, Bcl-2, and TP53 and targeted by 11 compounds including (+)-limonene, curzerene, furanodiene, germacrone, isoprocurcumenol, isocurcumenol, *β*-elemene, curdione, curcumol, procurcumenol, and demethoxycurcumin. Most of these compounds are the core ingredients listed in Table 2.

In the pharmacological evaluation, the results of the model group were consistent with those of many clinical studies (Ingelsson et al., 2007; Yao et al., 2017; Sun et al., 2021), indicating that the model was successfully created. CASR not only significantly improved the degree of myocardial fibrosis, inflammatory infiltration, and blood viscosity but also reduced the levels of IL-6, ET, TC, and LDL-C and increased the level of HDL-C. IL-6, and ET are related with vascular endothelial inflammation and functional damage (Hernandez et al., 2018). HDL-C can transport metabolic wastes, such as cholesterol in the blood, to the liver, thereby lowering cholesterol levels in the blood and reducing the occurrence of cardiovascular diseases. Arteriography showed that HDL-C levels were significantly negatively correlated with the degree of arterial stenosis (Yang et al., 2011). Therefore, HDL-C is an anti-atherosclerotic plasma lipoprotein cholesterol and a protective factor against CHD (Klancic et al., 2016). LDL-C is a risk factor for CHD, which transports lipids in the blood to vascular endothelial cells. Excessive lipid levels lead to excessive lipid deposition in vascular endothelial cells, aggravating the degree of atherosclerosis and increasing the risk of CHD (Holmes and Ala-Korpela, 2019). Studies have shown that statins can significantly reduce the level of LDL-C in patients and delay the progression of atherosclerosis, thereby inhibiting the progression of CHD. The results showed that the CASR high-dose administration group had similar therapeutic effect to the positive group. In summary, CASR can resist atherosclerosis and alleviate the progression of CHD. However, no differences in the levels of cTn-I, a biochemical marker of myocardial injury, were observed after administration, probably because it was less affected by CASR. The PI3K/AKT/mTOR is an important signal transduction pathway that regulates cardiac function (Wang et al., 2015). It can affect the occurrence and development of CHD by regulating inflammation, vascular endotheliogenesis, and cardiomyocyte apoptosis and by promoting glucose and lipid metabolism. Phosphatidylinositol 3-kinase (PI3K) is a lipid kinase that activates downstream targets. Protein kinase B (AKT) is a serine/threonine kinase that plays a key role in the regulation of cell metabolism, apoptosis, and vascular endotheliogenesis. B-cell lymphoma-2 (Bcl-2) is an oncogene and important regulator involved in the pathway of mitochondrial apoptosis. The tumor protein p53 (TP53) is a transcription factor that promotes apoptosis. The main function of nitric oxide synthase (NOS3) is to participate in the metabolism of arginine and proline and to catalyze the production of NO. Mammalian target of rapamycin (mTOR) is an atypical serine/threonine protein kinase that is mainly involved in many biological processes such as cell growth, proliferation, cell cycle, protein synthesis, and energy metabolism. The results of the RT-qPCR experiment preliminarily verified that CASR could regulate the PI3K/AKT/mTOR signaling pathway in CHD rats at the gene level.

**FIGURE 6:**
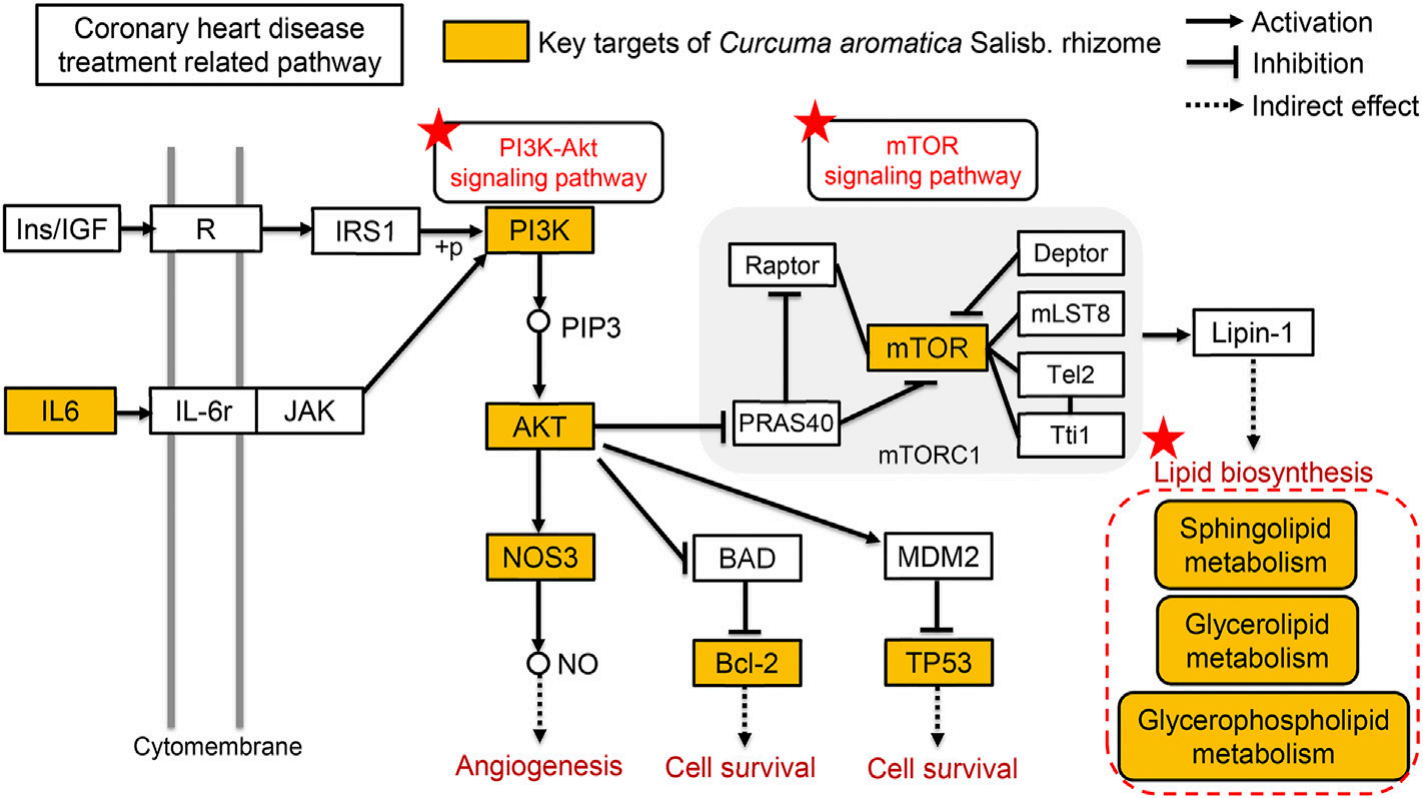
Comprehensive analysis of the therapeutic mechanism of CASR against CHD. The yellow squares represent the key targets of CASR in the treatment of CHD. Pentagrams represent key pathways involved. The arrows represent activation effects, the T-shaped arrows represent inhibition effects, and the segments represent indirect effects.

CASR was proved to improve dyslipidemia of CHD model. The present results showed that after treatment with CASR, the lipids recalled included SM, LPC, FA, and OxPC. Among these lipid compounds, the most significant callbacks were observed in SM 34:2; 2O, LPC 20:1/0:0, FA 20:5, and PC 32:0; 3O|PC 16:0_16:0; 3O, which are presumed to be biomarkers for the regulation of lipid metabolism in CHD by CASR. The metabolic network revealed that CASR plays a major role in CHD treatment through three metabolic pathways: sphingolipid metabolism, glycerophospholipid metabolism, and glycerolipid metabolism. Noteworthily, the sphingolipid signaling pathway is a shared pathway between network pharmacology and lipidomics results. Sphingolipids are key components of organelles and cell membranes, which regulate cell growth, differentiation, and senescence (Mendelson et al., 2014). They include ceramide, sphingomyelin, and sphingosine 1-phosphate. Ceramide is the core of sphingolipid metabolism, and sphingolipid metabolism revolves around its synthesis and decomposition (Airola and Hannun, 2013). The level of SM, which is a downstream decomposition product, increased significantly in the CHD model group and was significantly reduced after CASR administration, similar to previous reports (Sun et al., 2021). Furthermore, it was proved that CASR could significantly downregulate the expression level of SMS gene in the myocardium of rats with CHD. Sphingomyelin synthase (SMS) is the rate-limiting enzyme in the sphingomyelin metabolic pathway, according to the KEGG database analysis. By reshaping the structure of lipid rafts, SM plays a significant role in maintaining the function of lipid rafts and causing inflammation (Xue et al., 2019). Glycerophospholipid metabolism is closely related to inflammation and oxidative stress. This metabolic pathway contains a variety of enzymatic lipid metabolites, such as PC, which can be hydrolyzed by phospholipase A2 to form LPC and polyunsaturated fatty acids (PUFAs). PUFAs can be further oxidized to prostaglandins, thromboxanes, prostacyclins and hydroxylated fatty acids by cyclooxygenase, lipoxygenase and cytochrome P450 enzymes. These enzymatic lipid metabolites play important roles in inflammatory responses and lipid oxidation. In this study, it was found that the content of LPC in plasma of rats in the model group was significantly lower than that in the normal group, but it was called back after being treated with CASR (Yin et al., 2013; Dennis and Norris, 2015; Lu et al., 2017). In addition, Triglyceride metabolism has been found to be associated with CHD in genome-wide studies of CHD, and its metabolic enzymes are widely expressed in ventricular and myocardial cells, which may be related to ischemia (Zhang et al., 2020). Lipolysis determines the inflammatory process in the development of atherosclerosis (Hasham and Pillarisetti, 2006). Under the condition of normalizing PPAR-α expression, glycerol metabolism protects the heart from lipid deposition (Rame et al., 2011). In this study, it was found that CASR could improve the abnormal metabolism of triglyceride induced by CHD, which was speculated to be related to the protection of myocardial cells from lipid accumulation. Combined with the results of the pharmacological verification, a comprehensive illustration of the therapeutic mechanism of CASR against CHD is shown in Figure 6.

In addition, through correlation analysis, it was found that curzerene, isoprocurcumenol and (+)-curcumenol reduced blood viscosity, improved blood lipid levels, and reduced vascular endothelial damage. Furanodiene was responsible only for regulating blood lipid levels and vascular endothelial function. Curzerenone was only responsible for reducing blood viscosity. The above results also show that the CASR has multicomponent and multieffect characteristics.

## 5 Conclusion

In view of the unclear therapeutic mechanism of CASR against CHD, this study clarified that CHD regulates the PI3K/AKT/mTOR signaling pathway. This in turn regulates downstream sphingolipid metabolism and improves abnormal lipid levels and hemorheology. A reduction in vascular endothelial injury, inflammatory reactions, and lipid deposition was observed using UPLC-Q-TOF-MS/MS, network pharmacology, lipidomics, and RT-qPCR, in the treatment of CHD with CASR. In addition, curzerene, isoprocurcumenol, and (+)-curcumenol were identified as the main active compounds and are expected to be potential novel drugs for treating CHD. The results also showed the multicomponent and multipathway characteristics of CASR in the treatment of CHD.

## Data Availability

The original contributions presented in the study are included in the article/Supplementary Material, further inquiries can be directed to the corresponding authors.

## Glossary

CHD: Coronary heart disease
CASR: Curcuma aromatica Salisb. rhizome
ESI: electrospray ionization source
TIC: total ion chromatogram
TC: total cholesterol
TG: triglyceride
LDL-C: low-density lipoprotein cholesterol
HDL-C: high-density lipoprotein cholesterol
IL-6: interleukin-6
OxLDL: oxidized low-density lipoprotein
ET: endothelin
HFD: high-fat feed
ISO: isoproterenol hydrochloride
cTn-I: cardiac troponin I
MTBE: methyl tertiary-butyl ether
PFA: Paraformaldehyde
ADME: absorption, distribution, metabolism, and extraction
OB: oral bioavailability
DL: drug-likeness
Caco-2: human caucasian colon adenocarcinoma
BP: biological process: biological process
MF: molecular function: molecular function
CC: cellular component: cellular component
NC: normal control group
CASR-H: Curcuma aromatica Salisb. rhizome high-dose treatment group
CASR-L: Curcuma aromatica Salisb. rhizome low-dose treatment group
WBV: whole blood viscosity
EAI: erythrocyte aggregation index
H&E: hematoxylin and eosin
MT: Masson trichrome
GO: gene ontology
KEGG: Kyoto Encyclopedia of Genes and Genomes
BP: biological process: biological process
MF: molecular function: molecular function
CC: cellular component: cellular component
PI3K: phosphoinositide 3-kinase
AKT: protein kinase B
Bcl-2: B-cell CLL/lymphoma 2
TP53: tumor protein p53
NOS3: nitric oxide synthase 3
mTOR: mammalian target of rapamycin
QC: quality control
lysoPE: lysophosphatidylethanolamine
PE: phosphatidylethanolamine
PLS-DA: partial least-squares discriminant analysis
PCA: principal component analysis
SD: standard deviation
CAR: acylcarnitine
CE: cholesteryl ester
Cer: ceramide: ceramide
DG: diacylglycerol
HexCer: hexosylceramide
LPC: lysophosphatidylcholine
NAE: N-acyl ethanolamine
PC: phosphatidylcholine
PI: phosphatidylinositol
CASE: campesterol ester
SM: sphingomyelin
BA: bile acid
Cer: ceramide: ceramide
CL: cardiolipin
FA: free fatty acid; bileAcid, cholic acid
OxPC: oxidized phosphatidylcholine
SMS: sphingomyelin synthase
VIPd: variable importance in the projection
