# Supplementary material for: Therapeutic mechanism of Curcuma aromatica Salisb. rhizome against coronary heart disease based on integrated network pharmacology, pharmacological evaluation and lipidomics
Source: Front Pharmacol. 2022 Aug 9;13:950749. doi: 10.3389/fphar.2022.950749 (PMC9396035; doi:10.3389/fphar.2022.950749)
Supplement: Supplementary file 1 [file DataSheet1.docx]

Supplementary Material

**1. Supplementary File 1.** Materials and chemicals

High-fat feed (HFD; ingredients: 3% cholesterol, 0.5% sodium cholate, 0.2% propylthiouracil, 5% sucrose, 10% lard, and 81.3% basic feed) and normal feed were obtained from Jiangsu Xietong Pharmaceutical Bio-engineering Co. Ltd. (Jiangsu, China). Vitamin D_3_ injection (1 mL: 7.5 mg, 300,000 IU) was purchased from Shanghai General Pharmaceutical Co. Ltd. (Shanghai, China). Atorvastatin calcium tablets were purchased from Pfizer Pharmaceuticals Co. Ltd. (Liaoning, China). Isoproterenol hydrochloride (ISO, ≥ 99%) was purchased from Shanghai Aladdin Biochemical Technology Co. Ltd. (Shanghai, China). Sodium chloride injection (0.9%) was obtained from Shandong Qidu Pharmaceutical Co. Ltd. (Shandong, China). Paraformaldehyde (4% PFA) was obtained from the Beijing Labgic Technology Co. Ltd. (Beijing, China). ELISA kits for interleukin-6 (IL-6), oxidized low-density lipoprotein (OxLDL), endothelin (ET), and cardiac troponin I (cTn-I) were obtained from Shanghai Enzyme-linked Biotechnology Co. Ltd. (Shanghai, China).

Acetonitrile (ACN), methanol, and methyl tertiary-butyl ether (MTBE) (LC-MS grade) were purchased from Merck (Darmstadt, Germany). Ammonium formate, ammonium acetate, and isopropanol were purchased from ROE Scientific, Inc. (NY, USA). High-purity deionized water (18.2 MΩ) was obtained from a water purification system (Millipore, Bedford, MA, USA). Formic acid (LC-MS grade) used for the preparation of the mobile phase was purchased from Anaqua Chemicals Supply (Houston, TX, USA). SPE columns (LC-C_18_, 500 mg/mL) were purchased from ANPLE Scientific Instrument (Shanghai, China). All other chemicals used in this study were of analytical reagent grade.

Furanodiene, β-elemene, and demethoxycurcumin were obtained from the National Institutes for Food and Drug Control (Beijing, China); curzerene, germacrone, curcumenol, and isocurcumenol were purchased from Shanghai Yuanye Biotechnology Co., Ltd. (Shanghai, China); curdione and curcumin were purchased from Nanjing Liangwei Biological Technology Co. Ltd. (Nanjing, China). Furanodienon was purchased from Shanghai Shidande Standard Technical Service Co. Ltd. (Shanghai, China). Lyso PE (17:1), D5 TG (17:0-17:1-17:0), and PE (17:0) were purchased from Avanti Polar Lipids (Alabaster, AL, USA). The purity of all standards was > 98%.

TsingZol Total RNA extraction reagent (TSP401) was procured from Tsingke Biotechnology Co. Ltd. (Beijing, China). ChamQ Universal SYBR qPCR Master Mix (Q711-02/03) and HiScript II Q RT SuperMix for qPCR (+gDNA wiper) (R223-01) were procured from Vazyme Biotech Co. Ltd. (Nanjing, China); and the PCR primers were synthesized by Sangon Biotech Co. Ltd. (Shanghai, China).

##
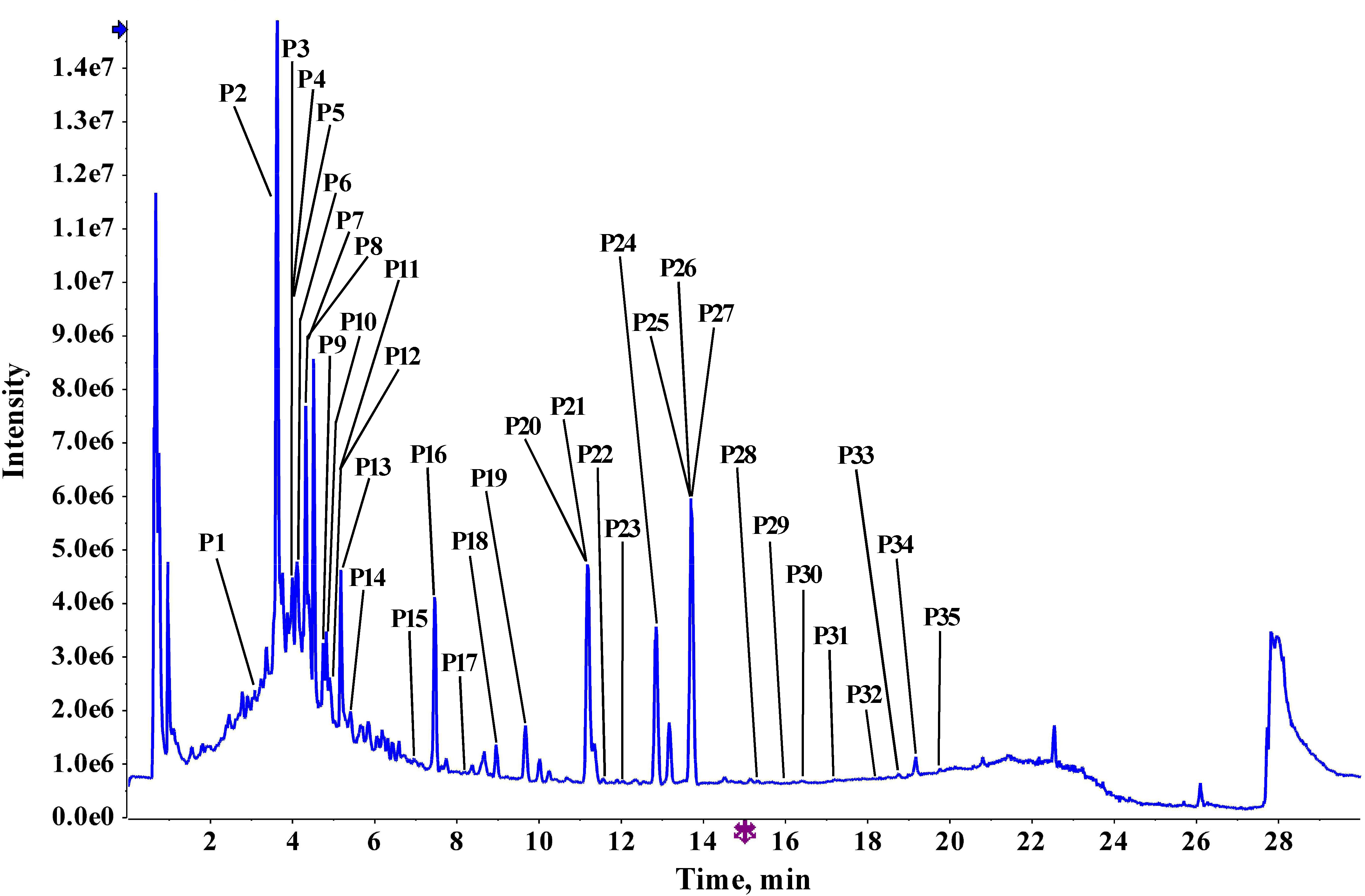
2. Supplementary Figures

**Fig. S1** Typical TIC chromatograms of CASR by UPLC-Q-TOF-MS/MS. P1 to P35 represent the compound number.





**Fig. S2** Effect of CASR on PI3K/AKT/mTOR signaling pathway. All values were presented as mean ± SD (n = 4). All *p*-values were calculated by T test. ^*^*p* < 0.05, ^⁎⁎^*p* < 0.01 compared with NC group, ^#^*p* < 0.05, ^##^*p* < 0.01 compared with M group. NC: normal control group; M: model group; P: positive drug (atorvastatin calcium tablets) control group; CASR: *Curcuma aromatica* Salisb. rhizome group.

**
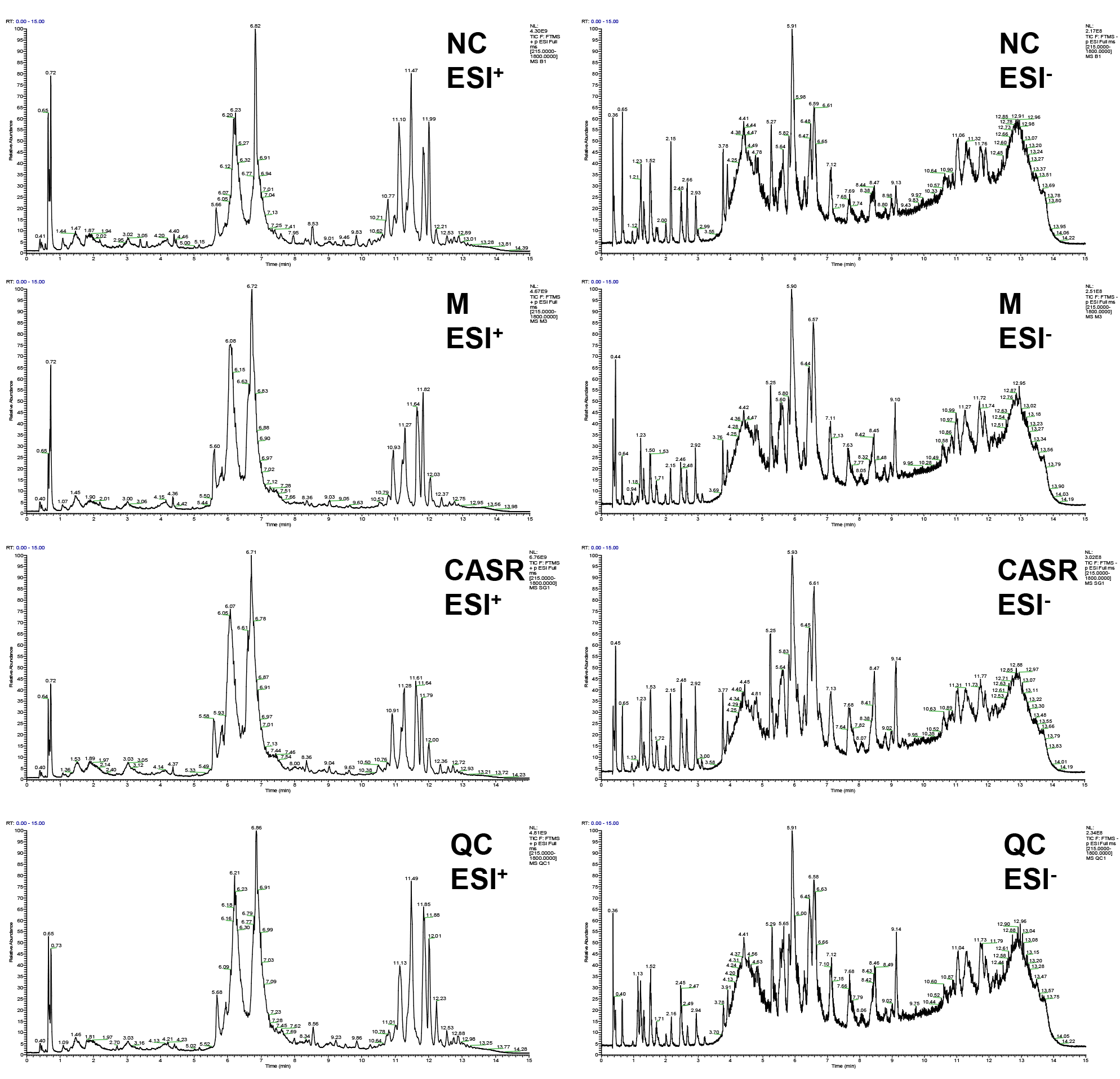
**

**Fig. S3** Typical TIC chromatograms of plasma lipids in positive and negative mode. NC: normal control group; M: model group; CASR: *Curcuma aromatica* Salisb. rhizome group. ESI^+^: positive mode; ESI^-^: negative mode.

**
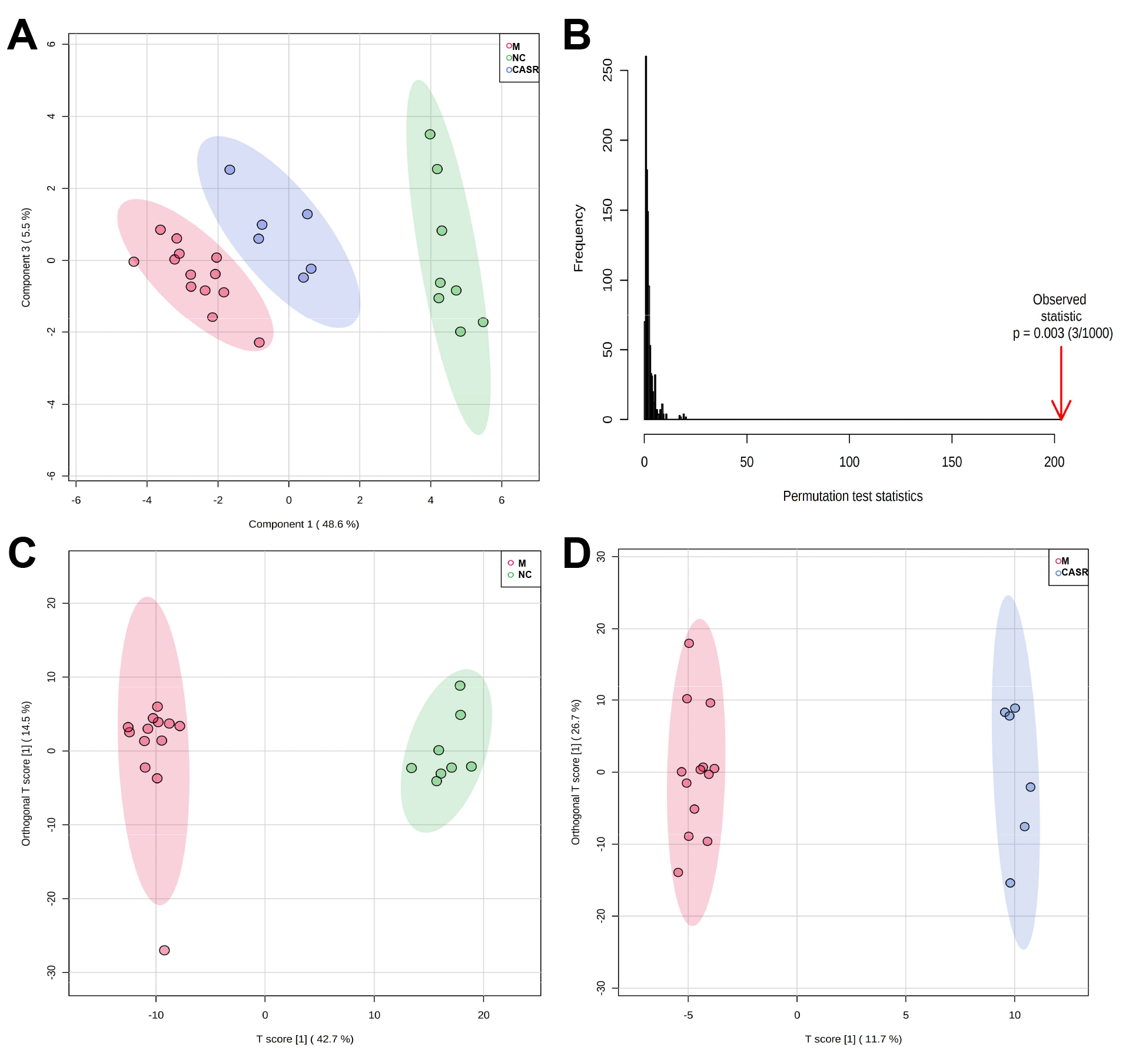
**

**Fig. S4** Metabolic profile analysis of lipids in positive mode. (A) PLS-DA plot; (B) Permutation test plots; (C) OPLS-DA plots of lipid metabolites in NC and M groups; (D) OPLS-DA plots of lipid metabolites in CASR and M groups. NC: normal control group; M: model group; P: positive drug (atorvastatin calcium tablets) control group; CASR: *Curcuma aromatica* Salisb. rhizome group.

**
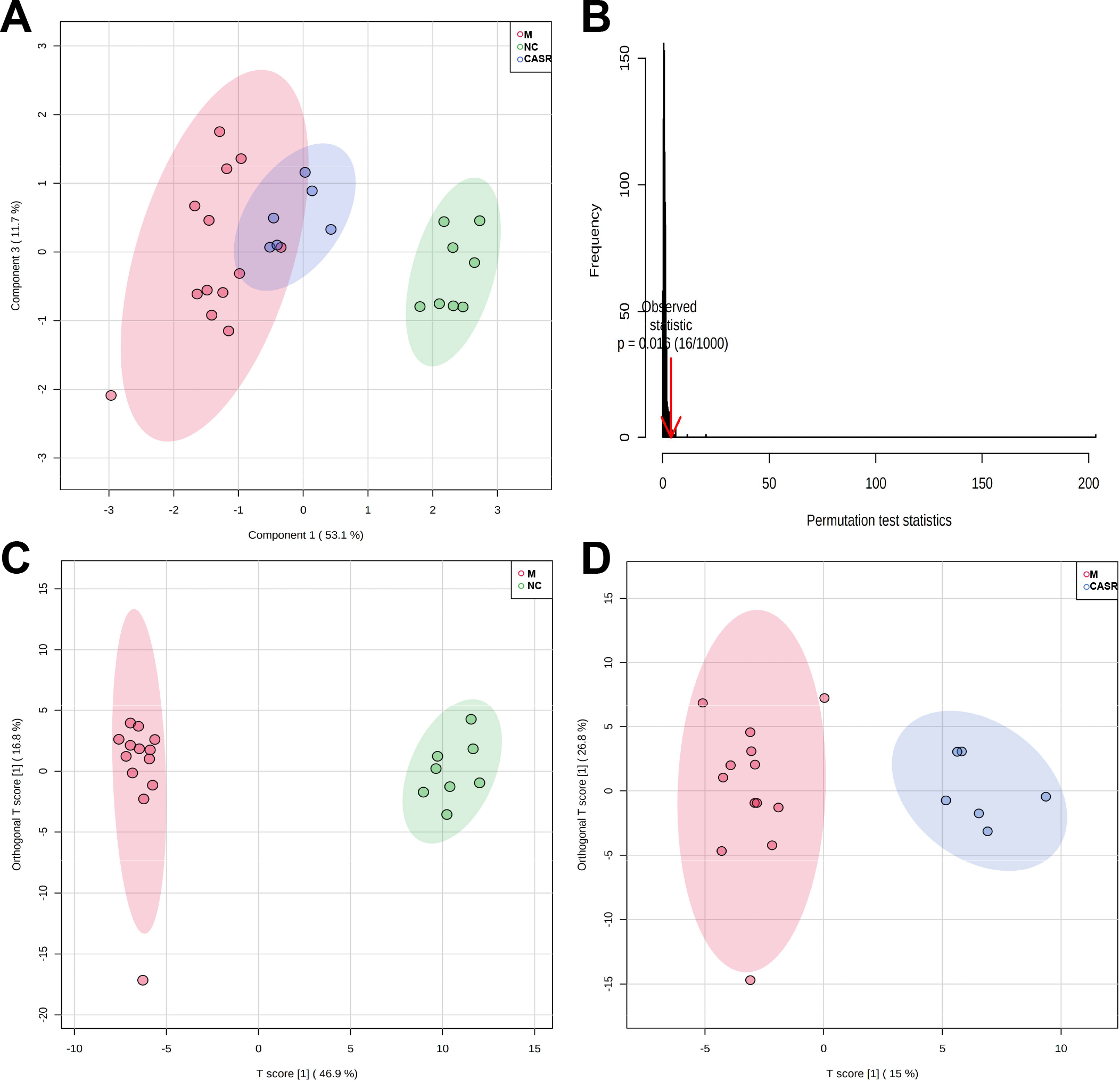
**

**Fig. S5** Metabolic profile analysis of lipids in negative mode. (A) PLS-DA plot; (B) Permutation test plots; (C) OPLS-DA plots of lipid metabolites in NC and M groups; (D) OPLS-DA plots of lipid metabolites in CASR and M groups. NC: normal control group; M: model group; P: positive drug (atorvastatin calcium tablets) control group; CASR: *Curcuma aromatica* Salisb. rhizome group.

## 3. Supplementary Tables

**Table S1** Primer sequences for RT-qPCR analysis.

| Gene | Forward sequences | Reverse sequences |
| --- | --- | --- |
| PI3K | ACAGGCACAACGACAACATCAT | AGGTAAGCCCTAACGCAGACAT |
| AKT | AGTCCCCACTCAACAACTTCT | GAAGGTGCGCTCAATGACTG |
| Bcl-2 | GAGGAACTCTTCAGGGATGGG | CTGTTGACGCTCTCCACACAC |
| TP53 | CCCCACCGCCTGTAAGATTC | GAGGGGTGGGGGATGGATA |
| NOS3 | CGACTATCCTGTATGGCTCTGAG | GATCCCCATTGCCAAATGTGC |
| mTOR | ACACCCTCCATCCACCTCAT | TAGCGGATATCAGGGTCAGGA |
| SMS | AGTGGGCGTTTTCTATTTGC | TACAGCGTGCCAACTATGC |
| β-actin | CCTCTATGCCAACACA | AGCCACCAATCCACACAG |

**Table S2** Compounds in CASR identified by UPLC-Q-TOF-MS/MS.

| No. | RT (min) | Compound | Formula | Mass | Ion Species | Theoretical Value (m/z) | Measured value (m/z) | ppm | MS/MS |
| --- | --- | --- | --- | --- | --- | --- | --- | --- | --- |
| P1 | 3.09 | Zedoalactone D | C_15_H_20_O_5_ | 280.1311 | [M+H]+ | 281.1384 | 281.1391 | 2.7 | 281.1395, 263.1313, 245.1181, 217.1218, 205.0881 |
| P2 | 3.71 | Gweicurculactone | C_15_H_16_O_2_ | 228.1150 | [M+H]+ | 229.1223 | 229.1225 | 0.9 | 229.1216, 211.1124, 196.0888, 195.0797, 183.1166 |
| P3 | 4.11 | Zedoalactone A | C_15_H_22_O_4_ | 266.1518 | [M+H]+ | 267.1591 | 267.1601 | 3.8 | 267.1618, 249.1501, 209.1170, 151.0750, 149.0958 |
| P4 | 4.15 | Zedoarolide B | C_15_H_22_O_5_ | 282.1467 | [M+H]+ | 283.1540 | 283.1546 | 2.0 | 265.1467, 219.1395, 205.1229, 201.1300, 181.1424 |
| P5 | 4.18 | 2, 3-Dehydro-1, 8-cineole | C_10_H_16_O | 152.1201 | [M+H]+ | 153.1274 | 153.1273 | -0.9 | 153.1268, 123.0771, 111.0835, 109.1008, 108.0541 |
| P6 | 4.25 | Zedoalactone C | C_15_H_22_O_4_ | 266.1518 | [M+H]+ | 267.1591 | 267.1597 | 2.2 | 267.1593, 249.1487, 209.1171, 151.0764, 109.0630 |
| P7 | 4.32 | Curcumenone | C_15_H_22_O_2_ | 234.1620 | [M+H]+ | 235.1693 | 235.1697 | 2.0 | 235.1698, 175.1124, 161.0965, 159.1164, 147.1159 |
| P8 | 4.39 | Wenjine | C_15_H_22_O_5_ | 282.1467 | [M+H]+ | 283.1540 | 283.1547 | 2.5 | 247.1306, 237.1490, 221.1488, 219.1371, 205.1241 |
| P9 | 4.74 | (1R,10R)-Epoxy-(-)-1,10-dihydrocurdione | C_15_H_24_O_3_ | 252.1725 | [M+H]+ | 253.1798 | 253.1799 | 0.4 | 253.1763, 235.1691, 107.0868 |
| P10 | 4.78 | Curzerenone | C_15_H_18_O_2_ | 230.1307 | [M+H]+ | 231.1380 | 231.1383 | 1.4 | 231.1377, 173.0956, 145.1017, 131.0838, 130.0789 |
| P11 | 4.82 | Isoprocurcumenol | C_15_H_22_O_2_ | 234.1620 | [M+H]+ | 235.1693 | 235.1698 | 2.4 | 235.1696, 217.1582, 193.1580, 177.1258, 175.1480 |
| P12 | 4.90 | Zedoalactone B | C_15_H_20_O_5_ | 280.1311 | [M+H]+ | 281.1384 | 281.1394 | 3.5 | 281.1429, 263.1235, 245.1186, 239.1264, 221.1193 |
| P13 | 5.17 | (4S,5S)-Germacrone 4,5-epoxide | C_15_H_22_O_2_ | 234.1620 | [M+H]+ | 235.1693 | 235.1700 | 3.2 | 235.1693, 217.1585, 193.1584, 175.1479, 149.0956 |
| P14 | 5.43 | Aerugidiol | C_15_H_22_O_3_ | 250.1569 | [M+H]+ | 251.1642 | 251.1647 | 1.9 | 251.1659, 233.1540, 165.0925, 145.0999, 119.0855 |
| P15 | 6.95 | Glechomanolide | C_15_H_20_O_2_ | 232.1463 | [M+H]+ | 233.1536 | 233.1539 | 2.3 | 233.1523, 175.1137, 157.1010, 147.0803, 142.0756 |
| P16 | 7.45 | Procurcumenol | C_15_H_22_O_2_ | 234.1620 | [M+H]+ | 235.1693 | 235.1696 | 1.5 | 235.1694, 217.1596, 189.1636, 177.1282, 161.0967 |
| P17 | 8.23 | (4S)-Dihydrocurcumenone | C_15_H_24_O_2_ | 236.1776 | [M+H]+ | 237.1849 | 237.1850 | 0.5 | 237.1797, 219.1729, 177.1227, 159.1378, 137.1333 |
| P18 | 8.95 | Neocurcumenol | C_15_H_22_O_2_ | 234.1620 | [M+H]+ | 235.1693 | 235.1698 | 2.4 | 235.1699, 217.1591, 199.1480, 189.1650, 175.1106 |
| P19 | 9.66 | 4-epi-Curcumenol | C_15_H_22_O_2_ | 234.1620 | [M+H]+ | 235.1693 | 235.1698 | 2.1 | 235.1725, 167.1070, 133.1020, 121.1013, 119.0857 |
| P20* | 11.27 | Furanodiene | C_15_H_20_O | 216.1514 | [M+H]+ | 217.1587 | 217.1589 | 0.9 | 217.1594, 161.0946, 147.0809, 133.1004, 117.0683 |
| P21* | 11.27 | (+)-Curcumenol | C_15_H_22_O_2_ | 234.1620 | [M+H]+ | 235.1693 | 235.1700 | 3.2 | 235.1709, 177.1282, 161.0956, 133.1009, 119.0853 |
| P22* | 11.51 | Demethoxycurcumin | C_20_H_18_O_5_ | 338.1154 | [M+H]+ | 339.1227 | 339.1236 | 2.5 | 339.1236 |
| P23* | 12.02 | Curcumin | C_21_H_20_O_6_ | 368.1260 | [M+H]+ | 369.1333 | 369.1321 | -3.1 | 369.1308, 335.1007, 299.0663, 285.1128, 253.0876 |
| P24 | 12.86 | Neocurdione | C_15_H_24_O_2_ | 236.1776 | [M+H]+ | 237.1849 | 237.1852 | 1.1 | 237.1861, 109.1005, 107.0856 |
| P25 | 13.72 | β-Elemenone | C_15_H_22_O | 218.1671 | [M+H]+ | 219.1743 | 219.1745 | 0.7 | 219.1758, 135.1165, 133.1046, 121.0981, 119.0831 |
| P26* | 13.72 | Curdione | C_15_H_24_O_2_ | 236.1776 | [M+H]+ | 237.1849 | 237.1858 | 3.9 | 237.1850, 125.0949, 109.0998, 107.0847 |
| P27 | 13.73 | (+)-Limonene | C_10_H_16_ | 136.1252 | [M+H]+ | 137.1325 | 137.1324 | -0.5 | 137.1324 |
| P28* | 15.30 | Curzerene | C_15_H_20_O | 216.1514 | [M+H]+ | 217.1587 | 217.1590 | 1.3 | 217.1591, 199.1448, 161.0973, 137.0961, 119.0853 |
| P29* | 16.01 | Furanodienon | C_15_H_18_O_2_ | 230.1307 | [M+H]+ | 231.1380 | 231.1383 | 1.4 | 189.1260, 185.1351, 149.0632, 143.0828, 140.9984 |
| P30 | 16.41 | Curcumol | C_15_H_24_O_2_ | 236.1776 | [M+H]+ | 237.1849 | 237.1850 | 0.5 | 219.1757, 201.1590, 135.1158 |
| P31* | 17.38 | Isocurcumenol | C_15_H_22_O_2_ | 234.1620 | [M+H]+ | 235.1693 | 235.1696 | 1.4 | 235.1691, 179.1103, 175.1479 |
| P32 | 18.28 | Curcumalactone | C_15_H_24_O_2_ | 236.1776 | [M+H]+ | 237.1849 | 237.1855 | 2.4 | 219.1747, 135.1174, 133.0255, 107.0878, 105.0709 |
| P33* | 18.74 | Germacrone | C_15_H_22_O | 218.1671 | [M+H]+ | 219.1743 | 219.1747 | 1.4 | 219.1736, 159.1157, 119.0856, 107.0859, 105.0693 |
| P34* | 19.48 | β-Elemene | C_15_H_24_ | 204.1878 | [M+H]+ | 205.1951 | 205.1954 | 1.5 | 121.1046, 107.0851 |
| P35 | 19.70 | δ-Elemene | C_15_H_24_ | 204.1878 | [M+H]+ | 205.1951 | 205.1958 | 3.5 | 205.1906, 149.1301, 135.1198, 107.0869, 105.0699 |

“*”identified with standard.

**Table S3** Differential lipids in plasma.

| **Compound name** | **m/z** | **RT (min)** | **Adduct ion** | **M *vs.* NC** | | | **CASR *vs.* M** | | |
| --- | --- | --- | --- | --- | --- | --- | --- | --- | --- |
|  |  |  |  | **P value** | **Fold change** | **VIP** | **P value** | **Fold change** | **VIP** |
| Cer 38:1;2O\|Cer 18:1;2O/20:0 | 594.5819 | 7.74 | [M+H]+ | 1.90E-10 | 11.06 | 1.43 | 3.95E-02 | 0.62 | 1.27 |
| Cer 42:0;4O\|Cer 18:0;3O/24:0;(2OH) | 666.6421 | 8.03 | [M+H-H_2_O]+ | 5.15E-09 | 3.33 | 1.39 | 5.56E-04 | 1.81 | 2.09 |
| DG 24:3 | 473.3195 | 4.35 | [M+Na]+ | 1.75E-05 | 0.51 | 1.25 | 1.89E-03 | 1.63 | 1.97 |
| DG 40:8\|DG 18:2_22:6 | 682.5389 | 6.38 | [M+NH_4_]+ | 1.52E-06 | 0.20 | 1.35 | 2.55E-02 | 0.51 | 1.46 |
| DG 50:0 | 843.7819 | 12.50 | [M+Na]+ | 4.43E-04 | 2.22 | 1.07 | 2.08E-02 | 1.72 | 1.59 |
| DG 52:1 | 869.7946 | 12.50 | [M+Na]+ | 5.74E-05 | 3.77 | 1.19 | 1.26E-03 | 2.63 | 2.02 |
| HexCer 36:1;2O\|HexCer 18:1;2O/18:0 | 728.6043 | 6.39 | [M+H]+ | 3.13E-09 | 48.92 | 1.39 | 2.84E-03 | 0.16 | 1.86 |
| LPC 14:0/0:0 | 468.3106 | 1.17 | [M+H]+ | 1.02E-04 | 0.55 | 1.13 | 7.70E-04 | 1.78 | 2.03 |
| LPC 20:0/0:0 | 552.4045 | 4.04 | [M+H]+ | 2.52E-05 | 0.69 | 1.18 | 5.33E-05 | 1.42 | 2.27 |
| LPC 20:1/0:0 | 550.3876 | 3.22 | [M+H]+ | 3.22E-05 | 0.62 | 1.19 | 8.14E-06 | 1.75 | 2.43 |
| LPC 20:4/0:0 | 544.3380 | 1.42 | [M+H]+ | 3.47E-05 | 0.62 | 1.20 | 6.78E-03 | 1.25 | 1.72 |
| LPC 22:4/0:0 | 572.3724 | 2.10 | [M+H]+ | 2.97E-05 | 0.59 | 1.20 | 2.18E-02 | 1.22 | 1.51 |
| LPC 24:1/0:0 | 606.4495 | 4.72 | [M+H]+ | 1.20E-05 | 0.62 | 1.22 | 1.81E-03 | 1.32 | 1.96 |
| NAE 18:2 | 324.2883 | 1.08 | [M+H]+ | 5.98E-06 | 0.52 | 1.28 | 1.59E-03 | 0.67 | 1.95 |
| PC 32:2 | 730.5399 | 5.61 | [M+H]+ | 3.16E-05 | 2.39 | 1.19 | 2.93E-02 | 1.47 | 1.41 |
| PC 36:0\|PC 18:0_18:0 | 790.6332 | 8.16 | [M+H]+ | 1.56E-04 | 1.90 | 1.11 | 1.64E-02 | 1.41 | 1.52 |
| PI 36:2 | 880.5857 | 5.66 | [M+NH_4_]+ | 9.63E-08 | 2.96 | 1.36 | 3.69E-04 | 1.55 | 2.11 |
| SM 32:1;2O | 675.5444 | 5.60 | [M+H]+ | 1.39E-07 | 3.22 | 1.36 | 1.44E-02 | 1.39 | 1.55 |
| SM 34:2;2O | 723.5388 | 5.34 | [M+Na]+ | 1.20E-04 | 2.30 | 1.13 | 1.42E-02 | 0.57 | 1.64 |
| SM 36:1;2O\|SM 18:1;2O/18:0 | 731.6064 | 6.56 | [M+H]+ | 5.43E-09 | 6.33 | 1.39 | 3.30E-02 | 0.71 | 1.20 |
| TG 52:0\|TG 16:0_18:0_18:0 | 880.8347 | 12.38 | [M+NH_4_]+ | 6.56E-06 | 2.19 | 1.25 | 3.51E-02 | 1.30 | 1.42 |
| TG 56:0\|TG 16:0_18:0_22:0 | 936.8883 | 12.74 | [M+NH_4_]+ | 7.21E-05 | 1.84 | 1.16 | 7.02E-04 | 1.60 | 2.12 |
| TG 58:0\|TG 16:0_18:0_24:0 | 964.9288 | 12.88 | [M+NH_4_]+ | 2.41E-05 | 1.68 | 1.20 | 2.44E-02 | 1.28 | 1.54 |
| TG 60:0\|TG 16:0_18:0_26:0 | 992.9557 | 12.97 | [M+NH_4_]+ | 4.51E-07 | 1.99 | 1.33 | 5.23E-03 | 1.39 | 1.82 |
| TG 60:1\|TG 16:0_18:0_26:1 | 990.9400 | 12.88 | [M+NH_4_]+ | 6.25E-05 | 2.12 | 1.16 | 4.46E-03 | 1.73 | 1.88 |
| TG 62:1\|TG 18:0_26:0_18:1 | 1018.9735 | 12.96 | [M+NH_4_]+ | 2.57E-05 | 1.94 | 1.20 | 7.18E-03 | 1.54 | 1.81 |
| TG 62:5\|TG 26:1_18:2_18:2 | 1010.9084 | 12.52 | [M+NH_4_]+ | 2.76E-04 | 0.47 | 1.12 | 4.31E-02 | 1.51 | 1.44 |
| TG 64:1\|TG 16:0_24:0_24:1 | 1047.0083 | 13.04 | [M+NH_4_]+ | 1.11E-03 | 1.84 | 1.01 | 2.36E-02 | 1.53 | 1.58 |
| CL 78:5\|CL 24:0_18:1_18:2_18:2 | 768.5494 | 5.99 | [M-2H]2- | 3.05E-05 | 1.41 | 1.16 | 4.48E-03 | 1.30 | 1.41 |
| FA 18:0 | 283.2644 | 2.48 | [M-H]- | 2.44E-05 | 0.79 | 1.13 | 1.89E-03 | 1.18 | 1.74 |
| FA 18:0;O | 299.2592 | 1.12 | [M-H]- | 8.29E-06 | 0.25 | 1.20 | 3.08E-02 | 1.72 | 1.19 |
| FA 18:3 | 277.2164 | 1.64 | [M-H]- | 2.62E-04 | 0.51 | 1.04 | 3.44E-02 | 1.48 | 1.18 |
| FA 20:5 | 301.2180 | 1.48 | [M-H]- | 6.99E-06 | 0.56 | 1.18 | 7.85E-04 | 1.73 | 1.76 |
| LPE 18:1 | 478.2950 | 1.71 | [M-H]- | 1.67E-05 | 1.97 | 1.16 | 1.07E-02 | 1.45 | 1.60 |
| LPE 18:2 | 476.2783 | 1.23 | [M-H]- | 4.75E-04 | 1.77 | 1.07 | 2.22E-02 | 1.41 | 1.39 |
| PC 32:0;3O\|PC 16:0_16:0;3O | 840.5757 | 5.82 | [M+CH3COO]- | 2.70E-08 | 1.99 | 1.33 | 4.93E-04 | 0.72 | 1.87 |
| PC 34:2;3O\|PC 16:0_18:2;3O | 864.5731 | 5.67 | [M+CH3COO]- | 9.94E-06 | 1.94 | 1.17 | 4.16E-03 | 0.65 | 1.82 |
| PC 34:2;3O\|PC 18:2_16:0;3O | 864.5737 | 5.41 | [M+CH3COO]- | 1.37E-04 | 1.35 | 1.09 | 4.44E-02 | 1.17 | 1.11 |
| PC 36:3;3O\|PC 18:1_18:2;3O | 890.5900 | 5.70 | [M+CH3COO]- | 5.18E-04 | 1.53 | 1.00 | 2.24E-02 | 0.72 | 1.51 |
| PC O-38:5\|PC O-18:1_20:4 | 852.6183 | 6.26 | [M+CH3COO]- | 1.14E-08 | 2.30 | 1.33 | 1.77E-02 | 0.74 | 1.42 |
| PI 36:3\|PI 18:1_18:2 | 859.5323 | 4.88 | [M-H]- | 1.81E-07 | 2.21 | 1.29 | 2.27E-02 | 1.33 | 1.25 |
| PI 38:4\|PI 18:1_20:3 | 885.5488 | 5.01 | [M-H]- | 2.88E-10 | 7.96 | 1.39 | 2.08E-02 | 1.48 | 1.33 |
| SM 42:3;2O | 869.6709 | 7.06 | [M+CH3COO]- | 1.91E-06 | 2.09 | 1.23 | 2.38E-02 | 1.31 | 1.54 |
| SM 44:2;2O | 899.7261 | 8.37 | [M+CH3COO]- | 3.52E-13 | 5.45 | 1.42 | 4.10E-02 | 0.85 | 1.10 |
